# Multi-PCM Lime Mortars Incorporating Polymer-Shell and Form-Stable Phase Change Materials for Energy-Efficient Building Envelopes

**DOI:** 10.3390/polym18121481

**Published:** 2026-06-12

**Authors:** Andrea Rubio-Aguinaga, Loucas Kyriakou, José María Fernández, Íñigo Navarro-Blasco, José Ignacio Álvarez

**Affiliations:** MATCH Research Group, Department of Chemistry, School of Sciences, University of Navarra, C/. Irunlarrea 1, 31008 Pamplona, Spain; arubioa@unav.es (A.R.-A.); lkyriakou@unav.es (L.K.); jmfdez@unav.es (J.M.F.); inavarro@unav.es (Í.N.-B.)

**Keywords:** phase change materials (PCMs), lime mortar, polymer-shell microcapsules, form-stable PCMs, multi-PCM, polymeric chemical additives

## Abstract

This study investigates the design and performance of lime mortars incorporating multi-phase change material (multi-PCM) systems as thermally responsive rendering materials for building-envelope applications under variable conditions. Moving beyond conventional single-PCM lime mortar approaches, this work proposes a controlled multi-PCM design framework in which a fixed total PCM dosage is distributed across selected phase-transition windows. Mortars combining PCMs with different transition temperatures (5–25 °C and 18–25 °C) were produced using two PCM types: silica-supported form-stable systems and polymeric-shell microencapsulated systems supplied as powders or aqueous slurries. All formulations contained 20% PCM and were optimized with polymeric additives, including a polycarboxylate ether-based superplasticiser and a starch-derived adhesion enhancer, to ensure suitable workability and applicability as rendering materials. Microstructural analyses showed that form-stable PCMs generated more heterogeneous pore structures, whereas polymeric-shell microencapsulated systems maintained pore structures similar to PCM-free mortars. Mortars containing metakaolin exhibited enhanced mechanical performance and durability, in some cases outperforming reference mortars, highlighting the importance of matrix refinement in the successful incorporation of multi-PCM systems. Thermal characterization revealed that form-stable systems produced broader phase transitions due to component interactions, while polymeric-shell microencapsulation preserved distinct transitions and enabled a wider, more controllable activation range. Under dynamic thermal conditions (−10 to 50 °C), all multi-PCM mortars demonstrated effective temperature buffering, achieving reductions of up to 1.5 °C during heating and 1.1 °C during cooling. Environmental and economic analyses highlighted that the benefits of PCM incorporation depend on matching PCM transition temperatures to specific climatic and application requirements. These findings position multi-PCM lime mortars as a promising route towards climate-adapted, thermally responsive renders with distributed and tailorable activation profiles.

## 1. Introduction

Phase change materials (PCMs) are capable of absorbing and releasing latent thermal energy during reversible phase transitions occurring within defined temperature ranges. Typically, involving solid–liquid transformations, these processes store and release energy through phase change enthalpy [1,2]. This latent heat storage capacity enables PCMs to function as thermal buffers under variable thermal conditions, attenuating temperature fluctuations through controlled energy exchange. As a result, PCMs have been widely investigated within latent heat thermal energy storage (LHTES) systems for thermal management applications [1,2,3].

Among the available PCM types, organic paraffinic compounds are most commonly used in building applications due to their chemical stability, relatively low cost, high latent heat, and reliable cycling performance [1,2,3]. In construction, PCMs can be incorporated at multiple scales, including macro-encapsulated systems (e.g., panels or containers), direct incorporation into mortars and plasters, microencapsulation within polymeric shells to prevent leakage, or as form-stable composites in which the PCM is physically confined within the porous matrix [3]. Each incorporation strategy directly influences not only thermal performance, but also material stability, compatibility, and applicability in building systems.

The effectiveness of a PCM is primarily governed by its phase transition temperature and latent heat capacity, which define both the operational temperature range and the amount of energy exchanged [4,5]. Consequently, PCM selection must be closely aligned with the climatic and operational conditions of the intended application [4,6,7,8,9,10,11]. Ensuring compatibility between the phase transition temperature and the thermal profile of the environment is essential for efficient latent heat utilization [4,6,7,8,9,10,11,12,13]. Additionally, long-term reliability depends on the ability of PCMs to withstand repeated thermal cycling without degradation [1,10,11,14,15].

Despite these well-established selection criteria, most PCM-modified mortars reported in the literature are designed around a single target temperature, typically associated with indoor comfort conditions or seasonal demands [15,16,17,18,19]. In such systems, a single PCM is incorporated with a phase transition temperature tailored to a predefined operating range [15,16,17,18]. While effective under stable conditions, this approach constrains the thermal response to a single activation threshold [20].

However, real thermal environments are inherently dynamic. Buildings are subjected to diurnal cycles, seasonal variations, and climate-dependent fluctuations that generate continuously shifting thermal profile [20,21,22,23]. These effects are further intensified by climate change, which is increasing temperature extremes, heatwave frequency, and inter-seasonal variability [24]. Under such conditions, single-PCM systems may only be activated during limited portions of the thermal cycle [20], leaving part of their latent heat storage potential underutilized and reducing overall thermal efficiency [4].

To address this limitation, research in latent heat thermal energy storage systems (LHTES) has demonstrated that combining multiple PCMs with different phase transition temperatures can significantly enhance thermal performance. Multi-PCM systems enable a more continuous and progressive activation of energy storage and release processes across a wider temperature range. Instead of concentrating phase change around a single temperature, this approach distributes thermal response over multiple intervals, reducing inactive periods and improving overall latent heat utilization [25,26].

Despite these advantages, the application of multi-PCM strategies in mortars remains limited [26]. Most studies have focused on optimizing PCM dosage, encapsulation methods, or mechanical compatibility, while the deliberate design of distributed phase transition profiles within a single material has received comparatively little attention [15,16,17,18,19,27]. This gap is particularly evident in lime-based mortars, where research has predominantly explored single-PCM systems [15,16,19,27].

The incorporation of multiple PCMs within a single mortar matrix represents a shift from a single-threshold to a distributed thermal response framework [20,26]. By extending the activation range, multi-PCM systems allow latent heat exchange to occur across broader temperature intervals [20,26], which is especially beneficial in climates with pronounced thermal variability. In such systems, each PCM contributes within a specific temperature range, resulting in complementary and sequential activation patterns where the system distributes energy absorption and release across multiple thresholds that better reflect real service conditions [20,26]. Importantly, this strategy is not aimed at increasing total PCM content, but at optimizing how thermal energy is stored and released across temperature ranges. Through careful selection of PCMs with distinct transition temperatures, the thermal response can be tailored without increasing overall dosage. In the context of lime-based rendering mortars, the novelty of this approach lies in transferring this distributed-response concept to a compatible mineral matrix while simultaneously evaluating the influence of PCM transition temperature, typology and incorporation format. Therefore, the present strategy moves beyond the conventional assessment of PCM addition as an isolated functional modification and establishes a comparative basis for the design of thermally tailored, cascaded or climate-adapted PCM lime renders.

Within this context, lime mortars provide a particularly suitable matrix for PCM incorporation. Their porous microstructure and chemical compatibility facilitate the integration of different PCM types, including microencapsulated and form-stable systems, with stable interfacial behaviour [15,28]. Compared to Portland cement, lime binders require lower calcination temperatures, resulting in reduced embodied carbon emissions [29,30]. Additionally, lime mortars reabsorb atmospheric CO2 through carbonation during service life, partially offsetting production-related emissions and improving their environmental profile [31,32,33].

Beyond sustainability considerations, lime mortars are highly relevant in retrofit applications, particularly for heritage and traditional buildings, where compatibility with existing materials is essential [34,35,36]. This is especially important in Europe, where approximately 85% of the building stock predates 2001, and 85–95% is expected to remain in use by 2050 [37]. Moreover, over 20% of buildings were constructed before 1945 and often exhibit poor thermal performance and elevated energy consumption [38]. These conditions highlight the importance of minimally invasive, compatible retrofit strategies that improve energy efficiency while preserving architectural and cultural value [38]. In this framework, enhancing the thermal performance of lime mortars through PCM incorporation represents a promising approach.

The present study proposes and evaluates multi-PCM lime-based rendering mortars for building envelope applications, incorporating two distinct PCMs within the same matrix to distribute the thermal response across selected phase-transition windows rather than concentrating it around a single transition temperature. These PCMs differ in phase transition temperature, typology (form-stable and microencapsulated), and physical format (powder and slurry). The experimental design is structured around three key variables: transition temperature distribution, PCM type, and incorporation format.

To isolate the effect of thermal design, both PCMs are incorporated at equal proportions (10% + 10% by weight of lime, bwol), ensuring that observed differences arise from phase transition distribution rather than total PCM content. The study investigates configurations covering both wide (5–25 °C) and narrower (18–25 °C) activation ranges, evaluating their impact on thermal behaviour and energy efficiency.

In addition to thermal performance, the environmental implications are assessed at the material level, considering the influence of PCM incorporation on the embodied carbon [4]. A comprehensive characterization is conducted, including fresh-state properties, microstructural analysis, and mechanical performance, ensuring that improvements in thermal functionality and sustainability are achieved without compromising material integrity or practical applicability.

## 2. Materials and Methods

### 2.1. Materials

The rendering mortars investigated in this study were produced using a hydrated calcitic air lime (CL90-S, Cal Industrial S.A., Pamplona, Navarra, Spain) as binder and a calcareous sand (CTH Navarra, Huarte, Spain) as fine aggregate. The hydrated lime exhibited an average particle size of approximately 10 µm, with less than 10% of particles exceeding 50 µm, while the calcareous sand presented a particle size distribution within the 0–1 mm range.

The chemical composition of both materials was determined by X-ray fluorescence (XRF) using a Bruker S2 Puma spectrometer (Bruker, Billerica, MA, USA) equipped with a silver-anode X-ray tube and operated under helium atmosphere with a 4 µm polypropylene filter. Quantification was performed using Spectra Results Manager software (Bruker AXS Spectra Elements v2.3). The hydrated lime was primarily composed of CaO (96.5 wt.%), with minor amounts of SO_3_ (1.3 wt.%), MgO (0.9 wt.%) and SiO_2_ (0.8 wt.%). The calcareous sand exhibited a composition of approximately 94.6 wt.% CaO, 2.3 wt.% SiO_2_, 0.8 wt.% Al_2_O_3_ and 0.7 wt.% MgO.

Mineralogical characterization was carried out by X-ray diffraction (XRD) using a Bruker D8 Advance diffractometer (Bruker, Billerica, MA, USA) with Cu Kα radiation (λ = 1.5406 Å). The hydrated lime was mainly composed of portlandite (Ca(OH)_2_), with calcite (CaCO_3_) as a secondary phase. The calcareous sand consisted predominantly of calcite, with minor amounts of quartz and phyllosilicate-type phases.

All mortar formulations were prepared with a constant binder-to-aggregate ratio of 21.7/78.3 by weight. The total water content was fixed at 25 wt.% of total solids in all mixtures to ensure direct comparability between formulations incorporating different phase change materials, eliminating variability associated with water dosage.

To achieve the required workability while maintaining constant water content, polymeric additives were considered: a polycarboxylate ether-based superplasticiser (MasterCast GT 205, Master Builders Solutions, Cornellà de Llobregat, Spain) was used to enhance flowability. Additionally, a starch-derived adhesion enhancer (Casaplast KO09 S, Nova Casanova, Barcelona, Spain) was incorporated to improve cohesion and substrate adherence. In selected formulations, metakaolin (MK, Metaver N, NEWCHEM, Baden, Austria) was added to promote pozzolanic reactions within the lime matrix. Its chemical composition, determined by XRF under the same conditions, was dominated by Al_2_O_3_ (49.3 wt.%) and SiO_2_ (45.4 wt.%), with minor amounts of MgO, K_2_O, Fe_2_O_3_, and Na_2_O.

A multi-PCM design strategy was adopted to evaluate the combined influence of phase transition temperature and encapsulation method on mortar performance. The selected PCM combinations were designed to cover different thermal activation windows: a broad transition range combining low- and high-temperature PCMs (5–25 °C), intended to extend the temperature interval over which latent heat storage may occur, and a narrower range combining intermediate- and high-temperature PCMs (18–25 °C), aimed at concentrating the thermal response within temperatures more closely associated with indoor comfort and mild-to-warm operating conditions. In this framework, the 5–25 °C combination was conceived as a broad-response strategy for building-envelope materials exposed to wider daily or seasonal temperature variations, where the low-temperature PCM can contribute under colder operational conditions, while the 25 °C PCM becomes active under warmer daytime or indoor-adjacent conditions. Conversely, the 18–25 °C combination was designed as a comfort-centered strategy, concentrating the phase-change contribution within a narrower temperature range associated with occupied indoor conditions and moderate heating or cooling demand. The comparison between these two combinations also provides a controlled framework for assessing multi-stage thermal regulation in lime-based renders, by evaluating whether latent heat exchange is more effectively distributed over a wider operational interval or concentrated within a comfort-related range. In this sense, the selected combinations provide an initial experimental basis for future cascaded or climate-adapted PCM render systems, in which sequential or partially overlapping phase transitions could be selected according to specific climatic profiles, façade exposure conditions or building-envelope operating scenarios.

Two main PCM types were considered: form-stable systems and microencapsulated systems. Within the latter, both aqueous dispersions (slurries) and dry powder microcapsules were used, enabling assessment of the effect of PCM format on dispersion within the lime matrix and its subsequent influence on mechanical, durability and thermal performance. To isolate these effects, the total PCM content was kept constant across all formulations and fixed at 20% bwol, distributed as 10% bwol + 10% bwol. This total dosage was selected based on previous optimization studies on lime-based mortars incorporating single PCMs at dosages between 5 and 20% bwol. These studies showed that 20% bwol provided the clearest and most application-relevant thermal response, particularly in terms of latent heat storage capacity and thermal buffering behaviour, while still preserving fresh-state consistency, hardened-state performance, mechanical response and suitability as rendering materials through adequate formulation adjustment [15,27,28]. Accordingly, the 10% bwol + 10% bwol distribution allowed two PCM phases to be combined while maintaining the total functional dosage established as the most suitable compromise between thermal performance and material feasibility. At the same time, this distribution ensured that the contribution of each individual PCM remained thermally relevant within the combined system, enabling a meaningful comparison between the different multi-PCM typologies and incorporation formats studied.

The form-stable PCMs (Rubitherm GmbH, Berlin, Germany) consist of paraffinic compounds embedded within a porous silica matrix that prevents leakage during phase transition. According to the manufacturer, the silica support has an average particle size of approximately 200 µm. Two materials were selected, with nominal melting temperatures of 5 °C and 25 °C. Differential scanning calorimetry (DSC) analysis (Section 2.2.6) revealed melting intervals of −3 to 7 °C and 13 to 26 °C, with peak temperatures at 6.0 °C and 24.6 °C, respectively. These materials are hereafter referred to as FS5 and FS25.

Microencapsulated PCMs in slurry form were supplied by MikroCaps (Ljubljana, Slovenia), with nominal transition temperatures of 5 °C, 18 °C and 25 °C. These consist of paraffin cores encapsulated within melamine-formaldehyde polymer shells and dispersed in water (approximately 35 wt.% PCM and 65 wt.% water). DSC analysis (Section 2.2.6) indicated melting intervals of 0–8 °C, 12–20 °C and 14–26 °C, with peak temperatures of 5.7 °C, 17.6 °C and 24.5 °C, respectively. These materials are denoted as MS5, MS18 and MS25. The water contained in the slurries was accounted for in the total mixing water to maintain the fixed water content of 25 wt.% of total solids.

Dry powder microencapsulated PCMs supplied by Microtek Laboratories, Inc. (Moraine, OH, USA) were also incorporated, with nominal melting temperatures of 18 °C and 24 °C. These consist of paraffin cores encapsulated within melamine-based polymer shells designed to prevent leakage during repeated thermal cycling. DSC analysis (Section 2.2.6) showed melting intervals of 8–19 °C and 16–26 °C, with peak temperatures at 16.0 °C and 24.2 °C. These materials are referred to as MP18 and MP25.

All PCMs used in this study operate through reversible solid–liquid transitions of paraffinic compounds, enabling latent heat absorption during melting and release during crystallization within defined temperature ranges. The different multi-PCM configurations adopted, together with their thermal activation ranges and encapsulation characteristics, are summarized in Table 1.

### 2.2. Methods

#### 2.2.1. Mortar Preparation

The preparation procedure was designed to ensure homogeneous dispersion of all solid constituents prior to water incorporation. Dry components, including air lime, calcareous sand, starch-based adhesion enhancer, metakaolin (when applicable), powdered PCMs (i.e., form-stable and dry microencapsulated systems), and an initial superplasticizer dosage of 0.25% by weight of lime (bwol), were first dry-mixed. This stage was carried out in a solid additive mixer (BL-8-CA, Lleal S.A., Granollers, Spain) for 5 min to promote uniform distribution of the constituents.

Subsequently, mixing water was added using a Proeti ETI 26.0072 mortar mixer (Proeti, Madrid, Spain). The total water content was fixed at 25 wt.% of total solids for all formulations. In mixtures incorporating slurry-based microencapsulated PCMs, the slurry was added simultaneously with the mixing water, and its intrinsic water content was accounted for by adjusting the externally added water to maintain a constant total water content. Mixing was performed at low rotational speed for 270 s to obtain a homogeneous fresh mortar.

The superplasticizer (SP) dosage was adjusted, when necessary, within a narrow range from 0.60 to 1.00% bwol. Small increments were progressively incorporated until suitable consistency for rendering application was achieved, defined by sufficient spreadability and stable adhesion on a saturated absorbent brick substrate.

The final optimised formulations are presented in Table 2. The nomenclature adopted reflects the PCM typology and the corresponding pair of melting temperatures, while the suffix “-MK” indicates the incorporation of metakaolin. The prefixes FS, MS and MP refer to form-stable systems, microencapsulated slurries, and microencapsulated powders, respectively, followed by the nominal phase transition temperatures of PCM1 and PCM2.

Fresh mortars were cast into specific geometries tailored to the corresponding test requirements. For thermal conductivity measurements, disc specimens with a diameter of 55 mm and thicknesses of 20 mm were prepared. Cylindrical specimens (30 mm in diameter and 40 mm in height) were produced for mechanical testing, following procedures established in previous studies [15,27]. For thermal efficiency evaluation, rectangular flat specimens (9 × 18 cm) with a thickness of 2 cm were moulded. All specimens were cured under controlled laboratory conditions at 20 ± 0.5 °C and 60 ± 5% relative humidity.

**Figure 1:**
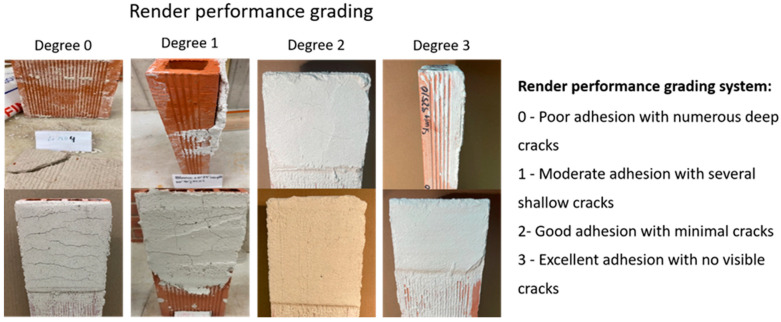
Qualitative visual grading scale for assessing render performance on saturated brick substrates, including representative examples of each grade.

Additionally, fresh mortars were applied as 0.5 cm thick render layers onto saturated brick substrates to assess their applicability as rendering materials. Adhesion performance and crack formation were evaluated through visual inspection using a qualitative grading scale previously developed for lime-based renders [28]. The grading criteria, along with representative examples for each performance level, are presented in Figure 1.

To halt the progression of carbonation and hydration reactions at specific curing ages (28, 91, 182, and 365 days), a freeze-drying protocol was applied. Specimens were first immersed in liquid nitrogen for 5 min to induce rapid freezing, followed by sublimation under vacuum conditions (1 Pa) for 24 h. This procedure effectively arrested further curing reactions while preserving the internal microstructure of the mortars without inducing additional phase transformations [15,27].

#### 2.2.2. Fresh State Tests

The fresh state behaviour of the mortars was systematically characterized through a series of standardised tests. Workability was assessed using the flow table method in accordance with UNE-EN 1015-3 [39], enabling evaluation of mixture consistency immediately after preparation. Fresh density was determined following UNE-EN 1015-6 [40], and the corresponding entrapped air content was calculated in compliance with UNE-EN 1015-7 [41]. Water retention capacity was evaluated according to UNE 83-816-93 [42]. Together, these tests provided a comprehensive characterization of the fresh-state properties of the mortars, allowing direct comparison between formulations incorporating different PCM typologies and metakaolin contents.

#### 2.2.3. Microstructural Analysis

Microstructural characterization was carried out to elucidate the influence of PCM typology and incorporation format on the internal structure of the lime mortars. Particular attention was given to differences associated with form-stable silica-supported PCMs and microencapsulated systems, as well as to the effect of incorporating microcapsules in powder or slurry form.

The pore structure of the mortars was analysed by mercury intrusion porosimetry (MIP) using a Micromeritics AutoPore IV 9500 porosimeter (Micromeritics Instrument Corp., Norcross, GA, USA), operating over a pressure range of 0.0015 to 207 MPa. Cubic specimens with approximate dimensions of 1 cm were extracted from the mortars and tested following the prescribed pressure program. All analyses were performed in duplicate to ensure reproducibility and representative microstructural assessment. MIP was used as a quantitative comparative technique to assess mercury-accessible porosity and pore size distribution under identical experimental conditions. Although widely established for porous construction materials, MIP does not directly represent the complete three-dimensional pore network or closed porosity. Accordingly, the results were interpreted together with SEM-EDS observations and related to the mechanical performance and durability response of the mortars.

Complementary microstructural observations were performed by scanning electron microscopy (SEM) using a COXEM EM-30N microscope (COXEM Co., Ltd., Daejeon, Republic of Korea). Imaging was conducted using both secondary electron (SE) and backscattered electron (BSE) detectors, enabling evaluation of surface morphology and compositional contrast. Elemental analysis was performed by energy-dispersive X-ray spectroscopy (EDS) using a Quantax Compact30 system (Bruker, Billerica, MA, USA), with spectral processing carried out using Esprit Compact software (Bruker, Billerica, MA, USA). Prior to SEM analysis, samples were sputter-coated with a thin layer of gold using a COXEM SPT-20 ion sputter coater to improve conductivity and minimise charging effects during imaging. For each formulation, two specimens were examined, and the selected micrographs illustrate the microstructural features consistently observed among the analysed replicates.

#### 2.2.4. Mechanical Strenght

Compressive strength testing was conducted to evaluate the development of mechanical properties over time and to assess the influence of different PCM typologies and incorporation formats on mortar performance. The tests were performed using a Frank/Controls 81565 hydraulic press (Karl Frank GmbH, Tiefenthal, Germany) equipped with a Proeti ETI 26.0052 compressive testing device (Madrid, Spain). Load was applied under controlled conditions at rates between 20 and 50 N/s, corresponding to total loading times of approximately 30 to 90 s. Measurements were carried out at curing ages of 28, 91, 182 and 365 days to monitor the evolution of mechanical behaviour. For each formulation and curing age, three cylindrical specimens were tested to ensure representative average values and experimental reliability.

#### 2.2.5. Durability Tests

Durability performance was assessed through accelerated ageing protocols designed to simulate critical environmental stress conditions associated with freeze–thaw cycling and salt crystallization. Freeze–thaw resistance was evaluated by subjecting the specimens to repeated cycles consisting of two consecutive stages. In the first stage, samples were fully immersed in water at ambient temperature for 24 h to ensure complete saturation. In the second stage, the saturated specimens were exposed to freezing conditions at −20 °C for an additional 24 h using a Samsung RZ80FJSW freezer. Each sequence constituted one cycle. The procedure was repeated for up to 28 cycles or until complete structural failure occurred, defined as total loss of material integrity. This protocol was adapted from EN 1367-1 (standardized methods for assessing freeze–thaw resistance) [43].

Salt crystallization resistance was assessed using magnesium sulfate (MgSO_4_) to induce crystallization pressures within the pore network. Specimens were immersed in a saturated MgSO_4_ solution at 20 °C and 95% relative humidity for 24 h, followed by oven drying at 110 °C for an additional 24 h. Rinsing was performed every 10 cycles to remove surface salt deposits and ensure accurate evaluation of progressive deterioration. The test was conducted for up to 28 cycles or until complete structural failure was reached. This procedure was adapted from EN 1367-2, which describes the magnesium sulfate test for evaluating resistance to salt crystallization [44].

For both durability protocols, cylindrical specimens (30 mm in diameter and 40 mm in height) were prepared in duplicate for each formulation. After each cycle, specimens were visually inspected to qualitatively document damage progression, and their mass was recorded to monitor weight changes associated with degradation. The severity of deterioration was classified using a predefined damage scale (Table 3), enabling systematic comparison between the different mortar formulations [15,45].

#### 2.2.6. Thermal Performance

Thermal conductivity (λ) was measured using a FOX50 Heat Flow Meter (TA Instruments, New Castle, DE, USA), equipped with two independently controlled Peltier plates that imposed a constant temperature gradient of 10 °C across the specimen. Measurements were conducted at −5 °C, 5 °C, 15 °C, 25 °C and 35 °C for all formulations, which were selected to cover a reasonable service temperature range for building envelope applications across diverse climatic scenarios, while also allowing the multi-PCM-bearing mortars to be evaluated under the principal thermal conditions expected during use. The selected temperature sequence therefore enabled thermal conductivity to be assessed across the relevant thermal states of the incorporated PCM combinations, including solid-state conditions, temperatures close to the phase-transition intervals, and temperatures above the transition ranges of both PCMs. This approach enabled a comprehensive thermal conductivity assessment across the different thermal states relevant to the intended application of the material.

Prior to testing, all specimens were cured for at least 28 days to ensure adequate microstructural stabilisation. The tests were conducted on disc specimens with diameter of 55 mm and thickness of 20 mm. For each formulation, three replicates were tested, and the results are reported as mean values with corresponding standard deviations.

Thermogravimetric analysis (TGA) of the raw PCMs was performed using a TA Instruments SDT650 simultaneous thermal analyser (TA Instruments, New Castle, DE, USA). Samples were heated from 35 to 1000 °C at a heating rate of 20 °C/min under an inert nitrogen atmosphere. Nitrogen was used as purge gas at a flow rate of 100 mL/min. The analyses were carried out to compare the thermal stability and decomposition behaviour of the different PCM typologies before their incorporation into the lime mortar matrix.

The thermal activation behaviour of the PCMs was characterized by differential scanning calorimetry (DSC) using a DSC25 apparatus (TA Instruments, New Castle, DE, USA)). Both individual PCMs and their corresponding binary mixtures (1:1 by weight, consistent with mortar compositions) were analysed to determine their effective activation ranges and potential interaction effects. Approximately 20 mg of powdered sample was placed in 40 µL aluminium crucibles and analysed under a nitrogen atmosphere (50 mL/min) with a purge flow of 400 mL/min. Two consecutive heating–cooling cycles were applied between −20 °C and 50 °C at a rate of 0.5 °C/min, selected to approximate realistic thermal conditions. Isothermal steps were included at the beginning and end of each cycle to ensure complete phase transitions and minimise residual thermal effects. Melting and crystallization temperatures, along with the corresponding enthalpies, were determined to characterize PCM behaviour.

The phase transition behaviour of PCM-containing mortars was also evaluated by DSC, including the determination of melting and crystallization temperatures and their associated enthalpies. For this purpose, the melting enthalpy (ΔH_m_), crystallization enthalpy (ΔH_c_), melting temperature (T_m_) and crystallization temperature (T_c_) were determined from three consecutive heating–cooling cycles between −20 °C and 50 °C, at a heating/cooling rate of 5 °C/min. Approximately 20 mg of sample, consisting of monolithic mortar fragments, were analysed to obtain a more representative assessment of the composite material. This approach enabled evaluation of the thermal energy storage and release capacity of the multi-PCM mortars.

**Figure 2:**
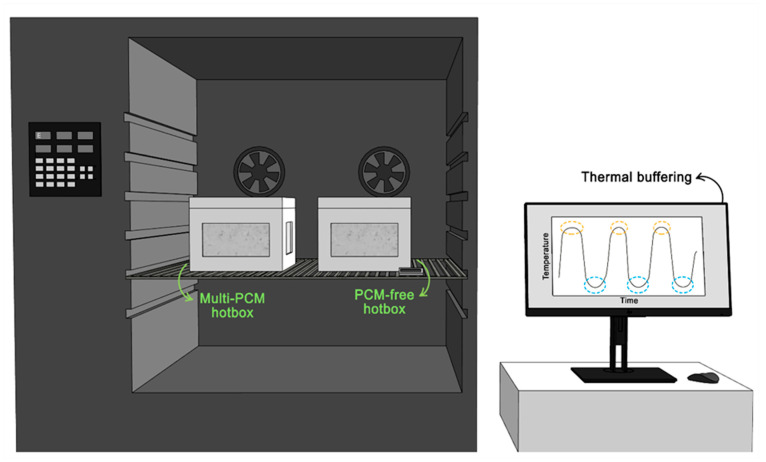
Experimental setup used to assess the energy efficiency of multi-PCM mortars under dynamic climatic conditions.

The thermal regulation capacity of the mortars was assessed using a hotbox experimental setup designed to reproduce controlled heating and cooling cycles over a broad temperature range relevant to building-envelope exposure. Hotbox-based methodologies have been widely used to evaluate the dynamic thermal response of PCM-containing mortars and building envelope components under laboratory-controlled thermal cycles [16,18,46,47]. Four flat mortar slabs (9 × 18 cm, 2 cm thick) were assembled within a thermally insulated expanded polystyrene enclosure and sealed with high-temperature-resistant silicone to ensure that heat transfer occurred predominantly through the mortar specimens (Figure 2). Two identical hotbox systems were placed inside a climatic chamber and subjected to cyclic temperature variations between −10 °C and 50 °C. One system contained multi-PCM mortar, while the other served as a reference with PCM-free mortar. Thermocouples installed inside the enclosures and within the climatic chamber continuously recorded temperature evolution during the cycles. This configuration provides an intermediate experimental level between intrinsic thermal characterization and full application-scale assessment, allowing the dynamic thermal response of the PCM-bearing mortars to be quantified under controlled and comparable conditions. In this way, the hotbox setup enables the potential of the mortars to buffer temperature variations, smooth thermal peaks and contribute to improved thermal comfort to be evaluated before progressing towards façade-scale testing, building simulations or long-term in situ monitoring.

The temperature difference between the multi-PCM and reference systems, ΔT(t), was monitored over time [15]. To quantify the overall thermal attenuation effect, the cumulative temperature-time response was calculated by integrating ΔT(t) over each cycle:

(1) $$Q^{*}=\int_{t_{1}}^{t_{2}}\Delta T\left(t\right)dt$$

where Q^∗^ (°C·s) represents the relative energy exchange and provides a comparative indicator of the dynamic thermal buffering capacity of the multi-PCM mortars under cyclic thermal exposure [15].

#### 2.2.7. Environmental and Cost Assessment

The environmental and economic performance of the mortars was evaluated through a cradle-to-gate carbon footprint analysis covering modules A1–A3 in accordance with EN 15804 [48], complemented by a material-level cost assessment. This approach quantifies the embodied impacts associated with raw material supply, transport and manufacturing processes prior to installation. The inventory data, calculation assumptions and detailed input values used for both the environmental and economic assessments are reported in Section 3.6, where the contribution of each component and process is further described.

Module A1 includes the extraction and processing of raw materials, encompassing lime production, aggregate processing, PCM manufacturing and the production of chemical admixtures [48]. Module A2 accounts for the transportation of all constituents from their respective production sites to the mortar preparation site [48]. In this study, Pamplona (Spain) was selected as the reference location for mortar preparation and potential application. This choice provides a representative case within a city with a significant built heritage, where lime-based materials are relevant for both ongoing conservation needs and future restoration interventions. Accordingly, transport distances were determined individually for each material based on its production origin and calculated with Pamplona as the destination point. Module A3 covers the mortar production stage, including mixing and preparation of each formulation [48].

For each mortar composition, the Global Warming Potential (GWP) associated with modules A1–A3 was quantified, allowing systematic comparison between formulations incorporating different PCM typologies and formats. The contribution of each component was calculated according to its proportion within the mixture, allowing identification of the relative carbon impact of binder, aggregate, PCMs and admixtures.

In parallel, an economic assessment was conducted considering raw material cost, transport and mixing processes. Raw material costs were obtained from supplier data to ensure industrial representativeness. Transport costs were estimated using typical road freight values (€/t·km), consistent with the routes defined in the environmental analysis. A representative transport cost of 0.18 €/t·km was adopted, in line with typical values for heavy-duty freight transport in Europe [49,50].

Mixing costs were calculated based on energy consumption, assuming a constant value of 0.200 kWh/m^3^ for all formulations, derived from industrial-scale data reported in the literature [51]. The electricity price was obtained from Eurostat for non-household consumers in Spain, adopting a value of 0.124 €/kWh based on the most recent available data [52].

The total cost of each formulation was calculated according to its composition, following an industrial-scale perspective representative of real construction scenarios. This approach ensures consistency between environmental and economic evaluations, providing a comparable framework for assessing both embodied carbon and cost at the material level.

## 3. Results and Discussion

### 3.1. Fresh State Characterization

The fresh-state properties of the optimised lime-based mortars are summarised in Table 4. The reference mixtures without PCM (LM and LM-MK) exhibited consistencies of 182 and 175 mm, respectively. Following PCM incorporation, consistency values ranged between 163 and 205 mm.

Despite this variation, no significant changes in workability were observed compared to the reference mortars, and all formulations maintained adequate consistency for rendering applications. This behaviour highlights the effectiveness of the optimization strategy adopted, particularly the adjustment of superplasticiser dosage and the use of an adhesion enhancer, which enabled the mixtures to retain suitable rheological properties despite PCM incorporation.

A similar trend was observed for fresh mortar density, with values ranging from 1.820 to 1.945 kg/L. The reference mortars exhibited the highest densities, while PCM-containing mixtures generally showed slightly lower values. This reduction is consistent with the lower density of the PCM phases compared to the mineral constituents of the lime matrix [53]. However, the differences were relatively small, and all formulations maintained comparable fresh densities, preserving the compactness and cohesion expected for lime-based mortars.

Entrapped air contents remained within a moderate range (2.0% to 4.4%), while water retention was consistently high across all formulations, varying between 93.3% and 96.5%. These results indicate that the incorporation of multi-PCM systems did not adversely affect the fresh-state stability of the mortars. High water retention is particularly beneficial for renders applied to absorbent substrates, as it helps maintain adequate moisture during application and early curing [54].

Importantly, these fresh-state properties were preserved despite the relatively high PCM content. Each formulation incorporated two PCMs simultaneously, reaching a total of 20% bwol. The ability to maintain fresh properties comparable to those of the reference mortars highlights the robustness of the optimization strategy and demonstrates that multi-PCM lime mortars can be designed without compromising practical workability.

**Figure 3:**
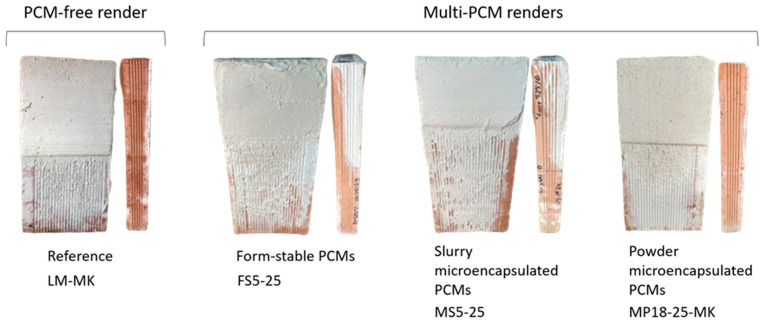
Representative render applications of optimised lime mortars on saturated brick substrates (front and side views). From left to right: LM-MK, FS5-25, MS5-25 and MP18-25-MK.

This satisfactory fresh-state behaviour was further confirmed by the qualitative rendering assessment (Table 4 and Figure 3). Most formulations achieved a performance grade of 3, corresponding to excellent adhesion, homogeneous coverage, and absence of visible cracking when applied to saturated brick substrates. Overall, these results demonstrate that, with appropriate formulation design, both microencapsulated and form-stable PCM systems can be successfully incorporated into multi-PCM lime mortars while preserving the fresh-state properties required for rendering applications.

### 3.2. Microstructural Studies

**Figure 4:**
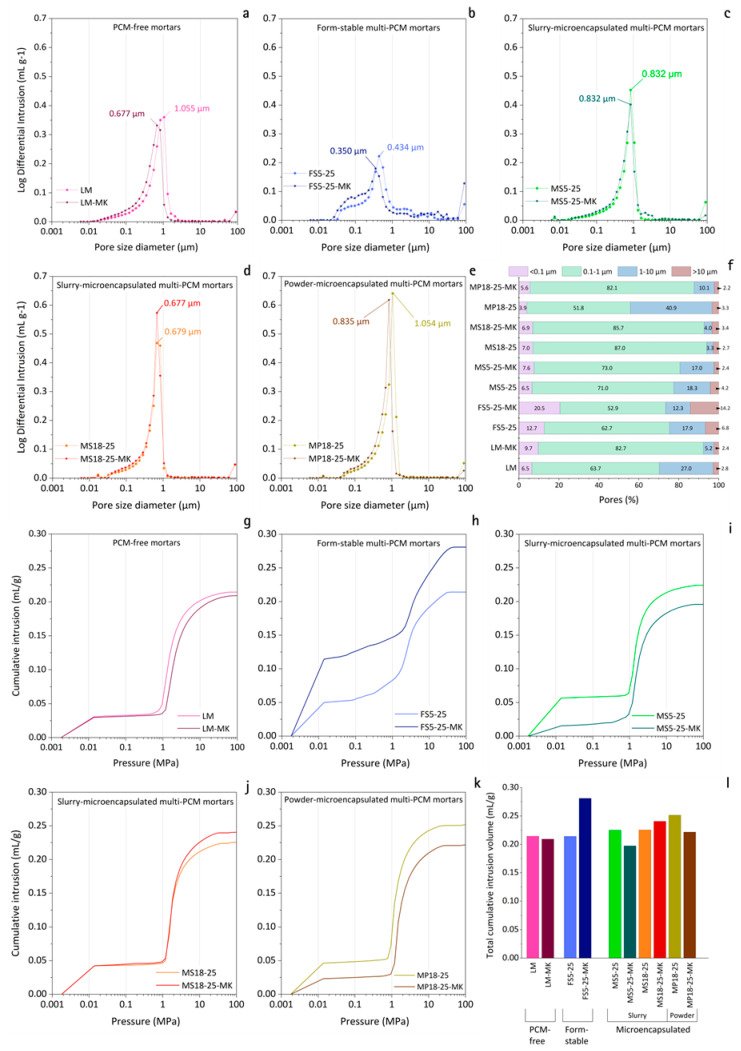
MIP-based pore structure characterization of lime mortars after 365 days of curing: (**a**) LM and LM-MK, (**b**) FS5-25 and FS5-25-MK, (**c**) MS5-25 and MS5-25-MK, (**d**) MS18-25 and MS18-25-MK, and (**e**) MP18-25 and MP18-25-MK pore size distributions; (**f**) pore volume distribution across different pore diameter ranges; (**g**) LM and LM-MK, (**h**) FS5-25 and FS5-25-MK, (**i**) MS5-25 and MS5-25-MK, (**j**) MS18-25 and MS18-25-MK, and (**k**) MP18-25 and MP18-25-MK cumulative intrusion curves; (**l**) total cumulative intrusion volume.

Mercury intrusion porosimetry (MIP) was performed to characterize the pore structure of the multi-PCM lime mortars and to assess the influence of PCM typology and incorporation format on the development of the porous network. The resulting pore size distributions are presented in Figure 4a–e, where the reference mortars are compared with the different multi-PCM configurations. This comparison enables evaluation of how the simultaneous incorporation of two PCMs, as well as their specific typology, affects the resulting microstructure.

To further quantify these distributions, pore volumes were classified into representative pore size ranges, as shown in Figure 4f. This complementary representation facilitates a clearer assessment of how the different PCM systems influence porosity across the main pore diameter intervals.

The reference mortars without PCM (LM and LM-MK), shown in Figure 4a, exhibit a well-defined unimodal pore size distribution. The dominant pore diameter is located at 1.055 µm for LM, while the incorporation of metakaolin shifts this maximum to 0.677 µm in LM-MK. This displacement towards smaller pore sizes reflects the well-established pore refinement associated with the pozzolanic reaction between metakaolin and calcium hydroxide [55,56]. The formation of additional calcium silicate hydrate (C-S-H) and related reaction products progressively densifies the microstructure, leading to a redistribution of the pore system towards finer diameters [55]. This effect may also be partially attributed to a filler contribution from metakaolin, which improves particle packing within the matrix [27,57,58].

Beyond this shift, both reference mortars remain characterized by a relatively narrow pore size distribution centred between approximately 0.1 and 1 µm, consistent with typical lime-based matrices [59]. This trend is further reflected in Figure 4f, where the incorporation of metakaolin leads to a significant reduction in the proportion of larger pores (1–10 µm), decreasing from 27.0% to 5.2%. Simultaneously, the fraction of pores between 0.1 and 1 µm increases from 63.7% to 82.7%, while pores smaller than 0.1 µm increase from 6.5% to 9.7%. This redistribution towards finer pores is characteristic of the microstructural densification associated with pozzolanic reactions [55,56,59,60].

**Figure 5:**
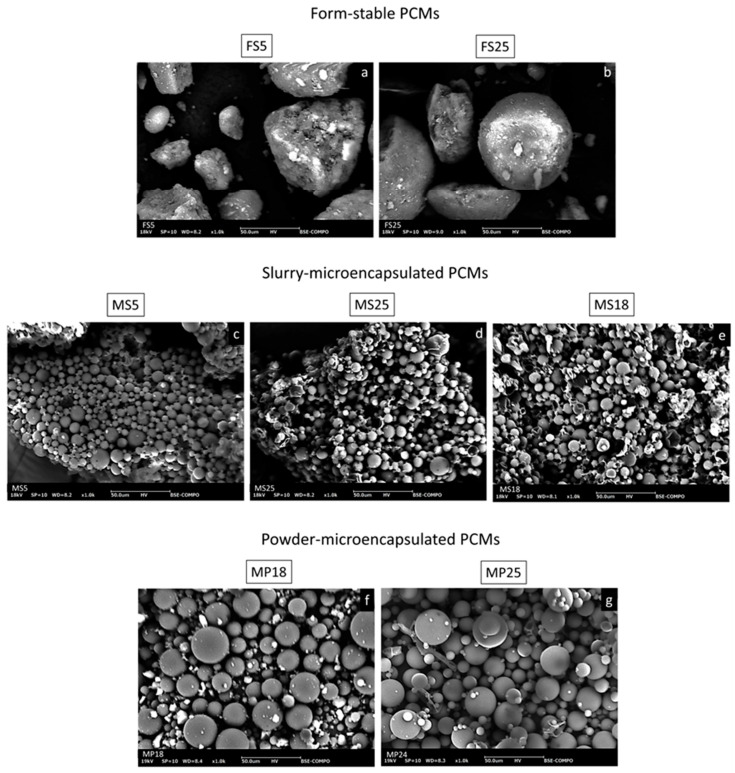
SEM micrographs (1000× magnification) of the raw PCMs used in the multi-PCM lime mortars: (**a**) FS5, (**b**) FS25, (**c**) MS5, (**d**) MS25, (**e**) MS18, (**f**) MP18, and (**g**) MP25.

**Figure 6:**
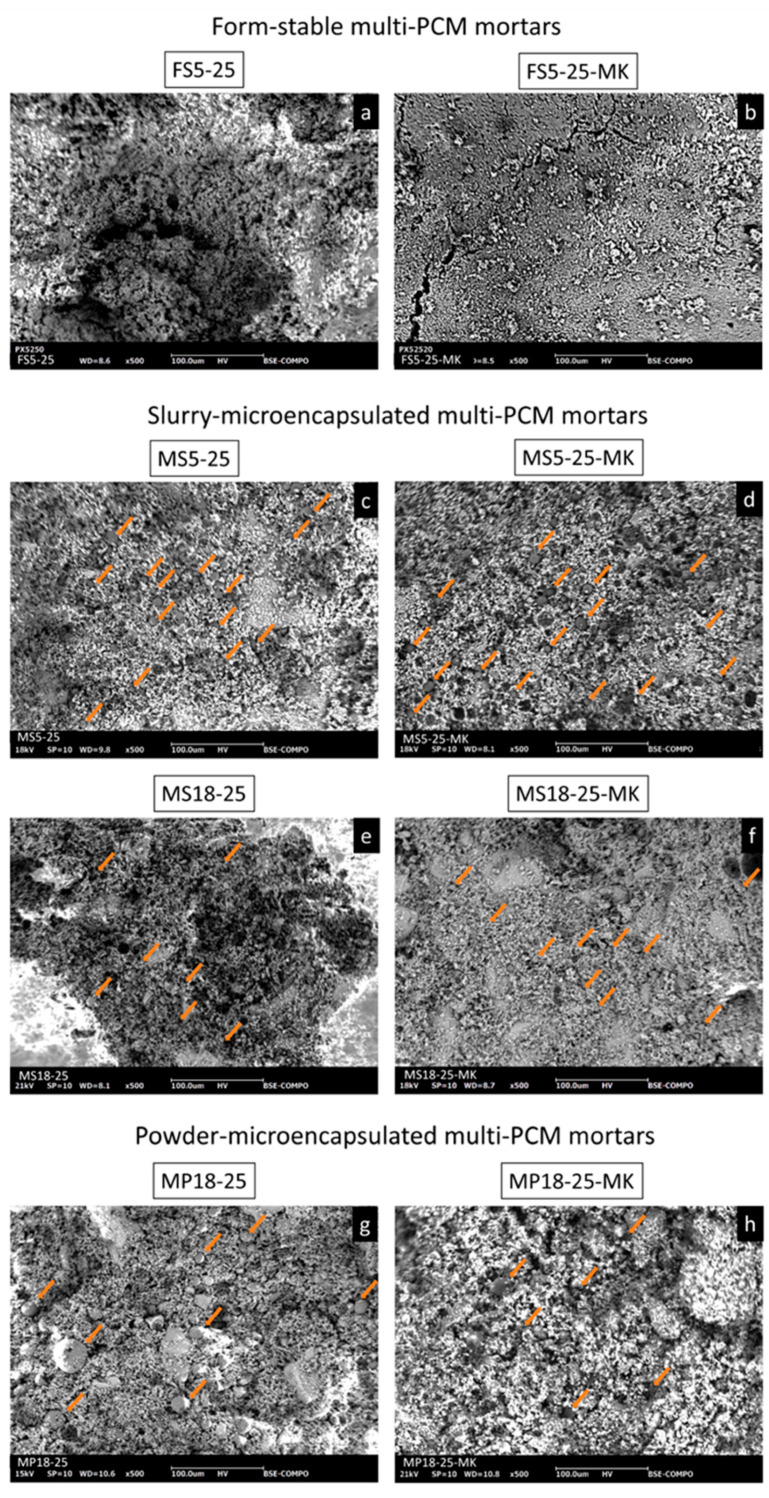
SEM micrographs (500× magnification) of the multi-PCM lime mortars: (**a**) FS5-25, (**b**) FS5-25-MK, (**c**) MS5-25, (**d**) MS5-25-MK, (**e**) MS18-25, (**f**) MS18-25-MK, (**g**) MP18-24, and (**h**) MP18-24-MK. Orange arrows indicate representative PCM inclusions within the lime matrix.

A distinct microstructural pattern is observed in mortars incorporating form-stable PCMs (Figure 4b). In these mixtures, the dominant pore diameter is reduced to 0.434 µm for FSS-25 and further shifts to 0.350 µm with the metakaolin addition. Although the main pore population is located at smaller diameters compared to the reference mortars, the overall distribution becomes significantly broader, with an extended range towards both finer and coarser pore sizes. This broadening indicates a more heterogeneous pore network, where multiple pore classes contribute simultaneously to total porosity. As supported by the scanning electron microscopy observations (Figure 5a,b), form-stable PCMs exhibit a wide particle size distribution (1–200 µm). Under these conditions, finer particles may contribute to a local filler effect, whereas coarser particles can generate interfacial transition zones or discontinuities within the lime matrix, promoting the formation of larger pores (as later supported by SEM observations, Figure 6a,b). This behaviour is further confirmed in Figure 4f, where form-stable mortars show a more dispersed pore size distribution compared to the reference systems. Notably, both the fraction of very fine pores (<0.1 µm) and large pores (>10 µm) increase significantly, reaching values of up to 20.5% and 14.2%, respectively. The simultaneous increase at both ends of the pore size spectrum reflects a substantially more heterogeneous and complex pore structure in form-stable multi-PCM mortars.

Mortars incorporating microencapsulated PCMs in slurry format (MS5-25 and MS5-25-MK) are presented in Figure 4c. The results indicate that the incorporation of slurry-based microencapsulated PCMs does not significantly alter the unimodal pore size distribution observed in the reference mortars (Figure 4a). For the MS5-25 system, both MK-free and MK-containing mixtures exhibit a dominant pore diameter of approximately 0.832 µm, suggesting that metakaolin addition does not modify the position of the principal pore population. However, a slight shift in the overall distribution towards smaller pore sizes is observed in the MK-containing mixture, reflected in an increased contribution of finer pores while maintaining the same peak position (Figure 4c). This behaviour suggests that the primary pore structure remains largely preserved, with the pozzolanic reaction subtly influencing the finer pore fraction [55,56]. This trend is further supported by the quantitative distribution shown in Figure 4f, where the fraction of pores in the 1–10 µm range decreases slightly (from 18.3% to 17.0%), while the proportion of pores below 0.1 µm increases (from 6.5% to 7.6%). Simultaneously, the dominant 0.1–1 µm range increases from 71.0% to 73.0%, indicating a modest redistribution towards finer pores consistent with limited pozzolanic activity.

Figure 4d shows the pore size distributions for mortars incorporating slurry-based microencapsulated PCMs with transition temperatures of 18 °C and 25 °C (MS18-25 system). Similar to the MS5-25 system, the unimodal pore structure is preserved (Figure 4c), with a dominant pore diameter of approximately 0.679 µm in the MK-free mortar and 0.677 µm after the metakaolin incorporation. This indicates that the main pore structure remains essentially unchanged. This behaviour is further reflected in the pore distribution shown in Figure 4f. However, the incorporation of metakaolin results in a slight increase in the fraction of larger pores and a marginal reduction in finer pores. This behaviour deviates from the expected pore refinement associated with pozzolanic reactions and suggests limited development of such reactions in this particular system under the studied conditions.

Figure 4e presents the pore size distributions of mortars incorporating microencapsulated PCMs in powder format. These systems also exhibit a predominantly unimodal pore structure. In the MK-free mortar, the dominant pore diameter (ca. 1.054 µm) closely matches that of the reference mortar (LM), indicating minimal structural disruption. Upon incorporation of metakaolin, the main peak shifts to 0.835 µm, reflecting the pore refinement trend observed in other systems. This effect is further confirmed in Figure 4f. The MK-free formulation shows a relatively high proportion of pores in the 1–10 µm range (40.9%), which decreases significantly to 10.1% after metakaolin incorporation. Consequently, the fraction of pores in the 0.1–1 µm range increases from 51.8% to 82.1%, while pores below 0.1 µm increase slightly (from 3.9% to 5.6%). These results confirm the pore-refining effect in the powder-microencapsulated systems.

To complement the pore size analysis, cumulative intrusion curves are presented in Figure 4g–l, providing insight into the progressive filling of the pore network and total accessible porosity. For the reference mortars (Figure 4g), both LM and LM-MK exhibit similar profiles, with a steep intrusion increase over a narrow pressure range corresponding to the dominant 0.1–1 µm pore domain (identified in Figure 4a). Final cumulative intrusion values (0.214 mL/g for LM and 0.209 mL/g for LM-MK; Figure 4l) indicate only minor changes in accessible porosity, consistent with the moderate pore refinement observed (Figure 4a).

In contrast, form-stable multi-PCM mortars (Figure 4h) exhibit more gradual intrusion curves extending over a wider pressure range, reflecting a broader distribution of pore sizes. This behaviour confirms the coexistence of both larger and finer pores, consistent with the heterogeneous pore structure identified in Figure 4b. The higher cumulative intrusion values (up to 0.281 mL/g for FS5-25-MK; Figure 4l) further indicate increased accessible porosity, partly associated with the intrinsic mesoporosity of the silica-supported PCM.

For slurry-microencapsulated systems (Figure 4i,j), the curves show a sharp increase within a limited pressure range, indicating a more homogeneous pore structure dominated by capillary pores. In the MS5-25 system (Figure 4i), metakaolin incorporation results in a slight reduction in cumulative intrusion (from 0.225 mL/g to 0.197 mL/g; (Figure 4l), consistent with modest pore refinement identified in Figure 4c. Conversely, in the MS18-25 system (Figure 4j), a slight increase in cumulative intrusion is observed (from 0.225 mL/g to 0.241 mL/g; Figure 4l), reflecting the increase in larger pores and the limited development of pozzolanic reactions (Figure 4d).

In powder-microencapsulated mortars (Figure 4k), the cumulative intrusion curves resemble those of the reference and slurry-microencapsulated systems, with a pronounced increase in the capillary pore range. The incorporation of metakaolin leads to a downward shift in the curve, indicating reduced accessible porosity. This is confirmed by the decrease in cumulative intrusion from 0.252 mL/g to 0.222 mL/g (Figure 4l), consistent with the significant pore refinement observed in Figure 4e.

Overall, the results presented in Figure 4 demonstrate that the incorporation of two PCMs within the same lime mortar matrix does not significantly alter the dominant pore size range, which remains primarily within 0.1–1 µm across all formulations. This highlights the effectiveness of the optimized formulations in accommodating multi-PCM systems without disrupting the characteristic capillary pore network of lime mortars. However, the PCM typology and format clearly influence the resulting microstructure. Form-stable systems produce broader and more heterogeneous pore distributions, while microencapsulated systems (both slurry and powder) maintain pore structures closer to the reference mortars. These differences are further reflected in the cumulative intrusion behaviour, indicating variations in accessible porosity and pore network complexity.

**Figure 7:**
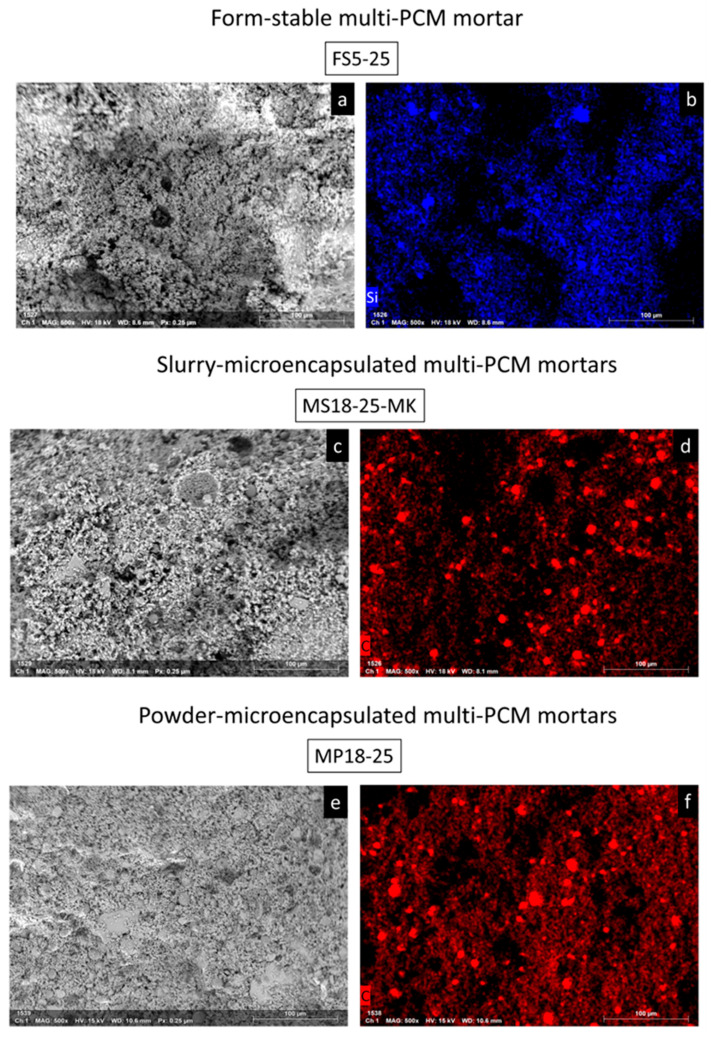
SEM micrographs and corresponding EDS elemental maps of representative multi-PCM lime mortars: FS5-25, (**a**) SEM micrograph and (**b**) Si mapping; MS18-25-MK, (**c**) SEM micrograph and (**d**) C mapping; and MP18-24, (**e**) SEM micrograph and (**f**) C mapping.

To complement the pore structure analysis obtained from MIP, scanning electron microscopy (SEM) was performed to further examine the intrinsic morphological characteristics of the raw PCMs (Figure 5) and their behaviour once incorporated into the multi-PCM lime mortars (Figure 6 and Figure 7).

SEM observations of the raw PCMs (Figure 5) reveal clear morphological differences between the three PCM typologies considered. The form-stable PCMs (FS5 and FS25) exhibit highly irregular particle morphologies, characterized by a broad particle size distribution and a combination of angular fragments and more rounded or partially spherical particles (Figure 5a,b). This heterogeneous morphology, together with the wide size range, supports the interpretation derived from MIP, where these systems displayed a more heterogeneous pore network. The coexistence of fine particles, which may locally contribute to a filler effect, together with larger particles capable of generating interfacial discontinuities within the lime matrix, is consistent with the simultaneous increase observed in both fine and coarse pore fractions.

In contrast, the slurry-microencapsulated PCMs (MS5, MS18 and MS25) are composed of well-defined spherical polymer-based microcapsules with smooth external surfaces and a relatively uniform size distribution (Figure 5c–e). Most microcapsules preserve their spherical geometry; however, some partially damaged or collapsed capsules can also be observed, which may be attributed to the sample preparation procedure for SEM, particularly the drying stage. Similarly, the powder-microencapsulated PCMs (MP18 and MP25) also exhibit a predominantly spherical morphology, consistent with their polymer-shell-encapsulated structure, although with a broader particle size distribution and generally larger capsule sizes compared to the slurry-based systems (Figure 5f,g).

Complementary SEM-EDS elemental maps of representative raw PCMs are provided in Figures S1–S3 (Supplementary Material) to support the chemical identification of the different PCM typologies before their incorporation into the lime mortar matrix. The FS5 system shows Si and O signals, consistent with the silica-based supporting matrix, together with a C signal associated with the organic PCM phase retained within the form-stable structure. In the slurry-microencapsulated MS18 system, the C and N signals are distributed across the microcapsule population, in agreement with the organic PCM phase and the nitrogen-containing polymeric shell. For the powder-microencapsulated MP18 system, the C elemental map further evidences the carbon-rich nature of the microencapsulated PCM particles. This complementary SEM-EDS characterization provides a direct elemental reference for each PCM typology, facilitating the subsequent interpretation of their distribution within the lime-based mortars.

SEM observations of the form-stable multi-PCM mortars (FS5-25 and FS5-25-MK) (Figure 6a,b) reveal a relatively heterogeneous matrix, in which local discontinuities can be identified in both formulations. This qualitative microstructural evidence was interpreted together with the quantitative MIP results, which showed a broader pore size distribution for these systems. The PCM phase cannot be clearly distinguished in the SEM micrographs, as its morphology does not present a well-defined contrast with respect to the surrounding matrix. However, EDS mapping performed on the MK-free mortar (Figure 7a,b) shows a homogeneous distribution of Si throughout the matrix, supporting the presence and relatively uniform distribution of the silica-supported form-stable PCM within the lime-based system. Thus, SEM and MIP provided complementary and consistent evidence of the microstructural features associated with the incorporation of the form-stable PCM systems.

SEM observations of the slurry-microencapsulated multi-PCM mortars (MS5-25 and MS18-25 systems) (Figure 6c–f) reveal a cohesive and continuous lime matrix, within which spherical microcapsules are clearly embedded. These microcapsules are distributed homogeneously throughout the mortar, as highlighted in the SEM micrographs, while preserving their structural integrity after incorporation into the lime matrix. This uniform dispersion is further confirmed by the EDS elemental mapping (Figure 7c,d), where the carbon signal appears evenly distributed across the matrix. The microcapsules are well integrated within the binder phase, with no evidence of significant clustering or segregation. This microstructural arrangement is consistent with the MIP results, which showed the preservation of a predominantly unimodal pore size distribution, indicating that the incorporation of slurry-based microcapsules does not significantly disrupt the capillary pore network of lime mortars.

Similarly, in the powder-microencapsulated multi-PCM mortars (MP18-24 system) (Figure 6g,h), a continuous lime matrix can be observed, within which spherical microcapsules remain structurally intact and homogeneously distributed. This spatial distribution is corroborated by the EDS mapping (Figure 7e,f), where the carbon map highlights the presence of microcapsules evenly dispersed across the matrix. In comparison with the slurry-based systems, the presence of larger microcapsules is more clearly identifiable, reflecting the broader particle size distribution associated with the powder format (Figure 5f,g).

Taken together, the SEM observations confirm that all PCM typologies are homogeneously distributed within the lime matrix, with no evidence of segregation or preferential accumulation. A more heterogeneous matrix can be observed in the case of the form-stable systems, where local discontinuities are present, in agreement with the MIP results. Nevertheless, the overall integrity of the matrix is preserved across all formulations. These observations highlight the effectiveness of the optimized formulation strategy, which enables the incorporation of different PCM systems while maintaining a coherent microstructural arrangement.

### 3.3. Mechanical Performance

**Figure 8:**
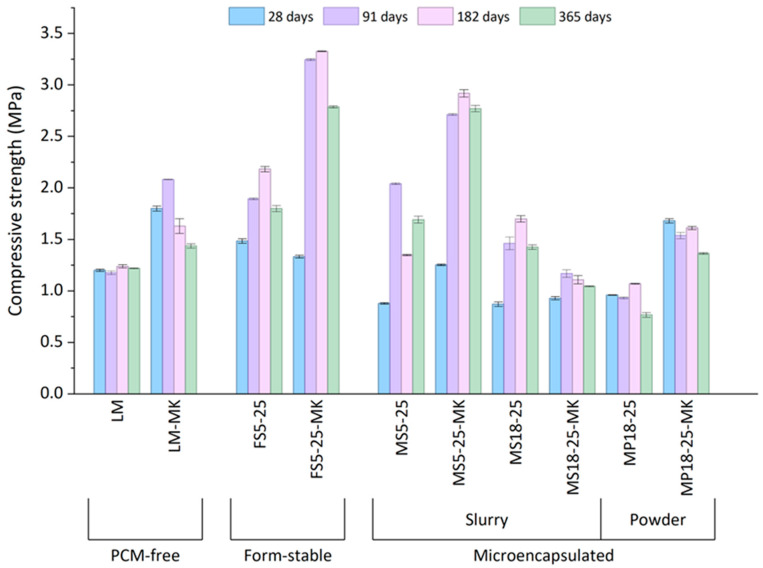
Evolution of compressive strength evolution of PCM-free and PCM-modified lime mortars incorporating different PCM typologies (form-stable and microencapsulated).

Figure 8 presents the compressive strength results of the multi-PCM lime mortars, highlighting distinct mechanical responses depending on the PCM typology. Among the investigated systems, mortars incorporating form-stable PCMs (FS5-25 and FS5-25-MK) exhibit the highest compressive strength values. This behaviour is particularly noteworthy given that MIP analysis revealed a broader and more heterogeneous pore size distribution for these formulations (Figure 4b). Rather than indicating a direct relationship between pore heterogeneity and strength loss, these results suggest that the compressive response of the form-stable PCM mortars is governed by the combined influence of pore structure, particle distribution and the mechanical contribution of the silica-supported particles within the lime matrix. SEM observations (Figure 7a,b) showed that the form-stable particles are relatively homogeneously dispersed and accommodated within the hardened matrix. This microstructural arrangement helps to explain why the broader pore network identified by MIP did not lead to a reduction in compressive strength. In these systems, the PCM is retained within a porous silica carrier, which behaves as a rigid mineral particulate phase within the lime matrix. The homogeneous distribution of these particles within the matrix allows the silica-supported structure to contribute to stress transfer under compressive loading, thereby favouring the mechanical response of the hardened mortar. Although previous studies often report reductions in mechanical performance following the incorporation of form-stable PCMs into construction materials [61,62], the present results demonstrate that, with appropriate formulation optimization, such systems can be successfully integrated without compromising compressive strength and may even enhance mechanical performance. In the present case, the higher compressive strength of FS5-25 and FS5-25-MK can therefore be rationalized by the combined effect of a relatively homogeneous particle distribution and the rigid mineral nature of the silica-supported framework, which together compensate for the more heterogeneous pore structure identified by MIP.

Within the microencapsulated systems, slurry-based formulations (MS series) generally exhibit higher compressive strength than their powder-based counterparts (MP series) (Figure 8). This behaviour can be attributed to differences in the nature of the microcapsules and their interaction with the lime matrix. Microencapsulated PCMs consist of polymeric shells that act as relatively compliant inclusions within the mineral matrix. Although SEM observations revealed a generally homogeneous distribution of microcapsules in both systems, the powder-based PCMs exhibited a wider particle size distribution, generally comprising larger capsules than the slurry-based formulations (Figure 5f,g). This characteristic could contribute to slightly lower mechanical performance, as larger compliant inclusions may locally reduce the efficiency of stress transfer within the hardened matrix.

In this context, slurry-based PCMs, comprising melamine–formaldehyde microcapsules dispersed in an aqueous medium, appear to generate a comparatively more favourable mechanical configuration within the lime matrix. By contrast, powder-based PCMs (MP series), introduced as dry melamine-based microcapsules, contain capsules of generally larger dimensions, which may produce mechanically softer regions of greater scale within the composite and therefore slightly reduce the overall load-bearing efficiency. This effect is particularly evident in the MP18-25 formulation without metakaolin, which exhibits compressive strength values of approximately 1 MPa, the lowest among all formulations studied (Figure 8). This behaviour is consistent with MIP results, which revealed a higher proportion of pores in the 1–10 µm range (Figure 4f), indicating a less favourable pore structure compared to the other multi-PCM mortars [63].

The influence of metakaolin (MK) is also apparent when comparing formulations with and without this pozzolanic addition (Figure 8). In most cases, MK incorporation results in higher compressive strength values. This trend aligns with MIP observations showing a shift in the pore system towards smaller diameters in MK-containing mortars (Figure 4), associated with the formation of secondary reaction products from the pozzolanic interaction between metakaolin and calcium hydroxide [55]. However, the magnitude of this effect remains moderate in the present study. MIP results indicate a general pore refinement without substantial microstructural transformation (Figure 4). This limited effect can be attributed to the curing conditions and mixture design, which are not optimised to maximise pozzolanic activity. More extensive reactions typically require higher metakaolin contents and curing environments with increased moisture availability [64]. Under the conditions adopted here, the pozzolanic contribution is therefore expected to be modest, consistent with both the observed pore refinement and the corresponding moderate increase in compressive strength. Notably, this trend is not observed for the MS18-25-MK formulation, where MIP analysis did not show clear evidence of pore refinement (Figure 4d). Accordingly, no significant improvement in compressive strength is observed compared to its MK-free counterpart (Figure 8), further supporting the correlation between microstructural densification and mechanical performance.

Overall, the compressive strength results indicate that the simultaneous incorporation of two PCMs within the same lime matrix does not adversely affect the mechanical performance of the mortars. In most cases, multi-PCM formulations exhibit compressive strength values comparable to, or in some instances higher than, those of the reference mixtures. This demonstrates that PCMs with different phase transition temperatures and typologies can be successfully integrated without compromising load-bearing capacity. These findings confirm that the optimised formulation strategy enables the incorporation of multi-PCM systems while preserving the microstructural integrity identified by MIP and SEM analyses (Figure 4 and Figure 6), as well as the mechanical performance required for rendering applications. All formulations satisfy the compressive strength requirements for lime-based rendering mortars, confirming their suitability for practical use [51].

### 3.4. Durability Evaluation

The durability of the multi-PCM lime mortars was assessed under two highly aggressive degradation conditions, freeze–thaw cycling and salt attack, due to their relevance in determining the long-term performance of these materials in real applications. Both exposure regimes are known to induce severe deterioration in lime-based systems, including internal stress development, loss of cohesion, and progressive disintegration [45,65]. Their evaluation is therefore essential to determine whether the incorporation of multiple PCMs can be achieved without compromising the durability of the mortar matrix.

**Figure 9:**
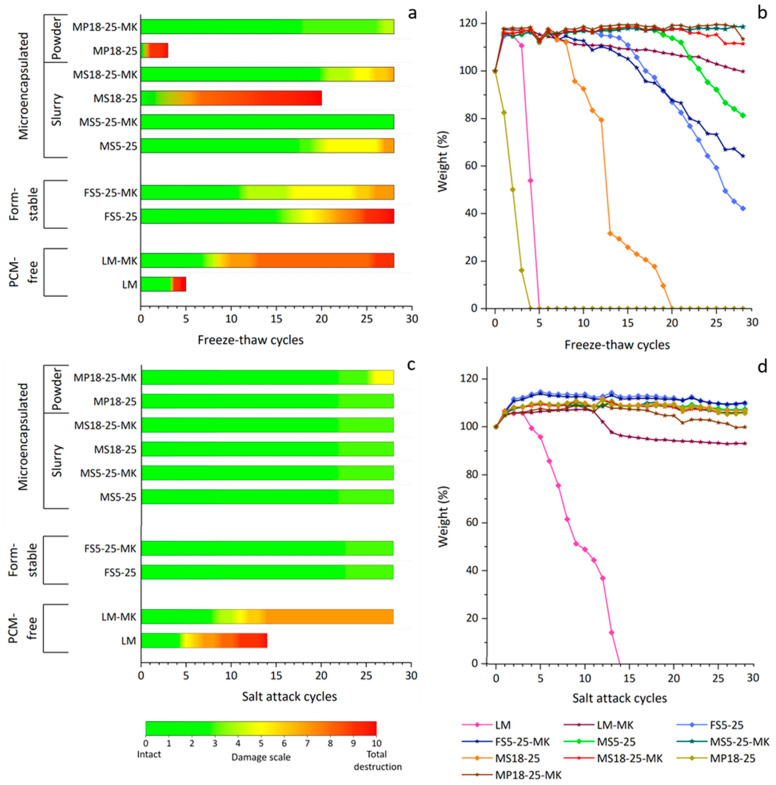
Durability performance of multi-PCM lime mortars: (**a**,**b**) freeze–thaw resistance, showing (**a**) damage progression according to the damage scale and (**b**) relative mass variation during cycling; (**c**,**d**) salt crystallization resistance, showing (**c**) damage progression according to the damage scale and (**d**) relative mass variation during cycling.

The freeze–thaw results (Figure 9a,b) highlight the critical role of both matrix composition and PCM typology in governing mortar durability. The reference mortar (LM) exhibits very limited resistance, failing after only 5 cycles (damage scale = 10), accompanied by rapid and significant mass loss (Figure 9b). The incorporation of metakaolin (LM-MK) leads to a marked improvement, with specimens surviving the full 28 cycles. However, the high damage level (damage scale = 9), together with measurable mass loss, indicates that substantial deterioration still occurs.

This behaviour is consistent with the moderate pore refinement observed in the MIP analysis (Figure 4a) and the corresponding increase in mechanical strength (Figure 8), which contribute to delaying failure but are insufficient to fully prevent damage under repeated freeze–thaw cycling.

When considering the multi-PCM systems, mortars incorporating form-stable PCMs (FS5-25) exhibit a durability response comparable to that of LM-MK, withstanding the full 28 freeze–thaw cycles but reaching a high damage level (damage scale = 9), accompanied by noticeable mass loss (Figure 9a,b). This behaviour is particularly noteworthy given that FS5-25 does not contain metakaolin, suggesting that silica-supported PCMs can partially compensate for the absence of matrix refinement in terms of resistance to complete failure. However, the elevated damage level indicates that this improvement remains limited.

**Figure 10:**
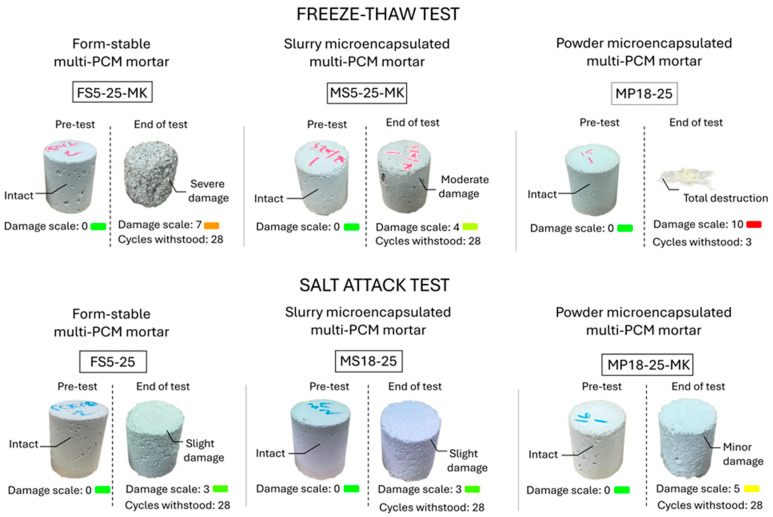
Representative specimens of multi-PCM lime mortars showing initial and final conditions after freeze–thaw and salt crystallization tests.

This response is consistent with MIP results which showed that these formulations present a broader and more heterogeneous pore size distribution compared to the reference mortars (Figure 4b). Such heterogeneity implies a less uniform pore network, which may facilitate progressive deterioration under repeated freeze–thaw cycling [66]. The addition of metakaolin (FS5-25-MK) reduces the damage level to 7 and results in a more stable mass evolution (Figure 9b), as also illustrated in Figure 10. This indicates that the microstructural refinement provided by metakaolin contributes to mitigating degradation, although the improvement remains moderate.

A different behaviour is observed in mortars incorporating microencapsulated PCMs in slurry form (MS systems), which generally exhibit enhanced freeze–thaw resistance compared to PCM-free references (Figure 9a). In all cases, slurry-based PCM incorporation increases the number of cycles withstood and reduces the final damage level, accompanied by a more stable mass evolution. This indicates a beneficial effect of PCM inclusion under freeze–thaw cycling.

The addition of metakaolin further enhances performance (Figure 9a). In the MS5-25 system, the damage level decreases from 7 to 4 upon MK incorporation, while in the MS18-25 system, MK enables the material to withstand the full 28 cycles, compared to failure at 20 cycles in the MK-free formulation, with reduced mass loss (Figure 9b). When comparing MK-free systems, MS5-25 clearly outperforms MS18-25, surviving all 28 cycles with moderate damage (damage scale = 7), whereas MS18-25 fails earlier and exhibits more pronounced degradation (damage scale = 10). A similar trend is observed for MK-containing systems, where MS5-25-MK shows lower damage (damage scale = 4) than MS18-25-MK (damage scale = 7). This trend is consistent with the presence of the low-temperature (5 °C) PCM in the MS5-25 formulation, whose phase transition temperature is closer to the freezing range and could contribute to moderating local thermal fluctuations during cycling [67,68,69].

For mortars incorporating microencapsulated PCMs in powder form (MP systems), the MK-free formulation (MP18-25) shows poor freeze–thaw resistance, failing after only 3 cycles (damage scale = 10) with significant mass loss (Figure 9a,b), as also evidenced by the complete disintegration observed in Figure 10. This behaviour is consistent with its lower mechanical strength (Figure 8) and with the MIP results, which revealed a higher proportion of pores within the 1–10 µm range for this formulation. In contrast, the incorporation of metakaolin (MP18-25-MK) results in a substantial improvement, allowing the material to withstand the full 28 cycles with low damage (damage scale = 4) and stable mass evolution (Figure 9a,b). This performance is comparable to that of the best-performing multi-PCM systems, indicating that the limitations associated with powder PCM formats can be effectively mitigated through matrix refinement.

Durability under salt attack provides complementary insights into resistance to crystallization-induced damage (Figure 9c,d). The reference mortar (LM) exhibits limited resistance, failing after 14 cycles (damage scale = 10) with significant mass loss. The incorporation of metakaolin (LM-MK) improves performance, allowing the material to complete 28 cycles with reduced damage and more stable mass evolution.

All multi-PCM mortars show consistently favourable behaviour under salt attack, completing 28 cycles with low damage levels (typically around 3) and stable mass evolution. This response is observed regardless of PCM typology or format, including both form-stable and microencapsulated systems. Unlike freeze–thaw conditions, resistance to salt-induced degradation appears to be less sensitive to PCM characteristics and more dependent on the overall pore structure of the mortar. In all cases, the pore network appears compatible with a stable response to crystallization processes, as confirmed by the minor surface damage observed in representative specimens (i.e., FS5-25, MS18-25 and MP18-25-MK) (Figure 10).

Overall, the results demonstrate that multi-PCM lime mortars exhibit satisfactory durability under both freeze–thaw and salt attack conditions. While salt attack remains consistently high across all formulations, freeze–thaw performance is strongly influenced by microstructural characteristics, particularly pore structure and PCM dispersion. These findings highlight the importance of formulation optimization, combining appropriate PCM typologies with adequate matrix refinement, as a key strategy to ensure the long-term durability of multi-PCM lime mortars. In addition, future application-scale studies could complement these durability indicators with long-term monitoring frameworks integrating environmental exposure and material-response data, to follow the service performance of PCM-enhanced envelope systems under variable conditions [70,71].

### 3.5. Thermal Behaviour

**Figure 11:**
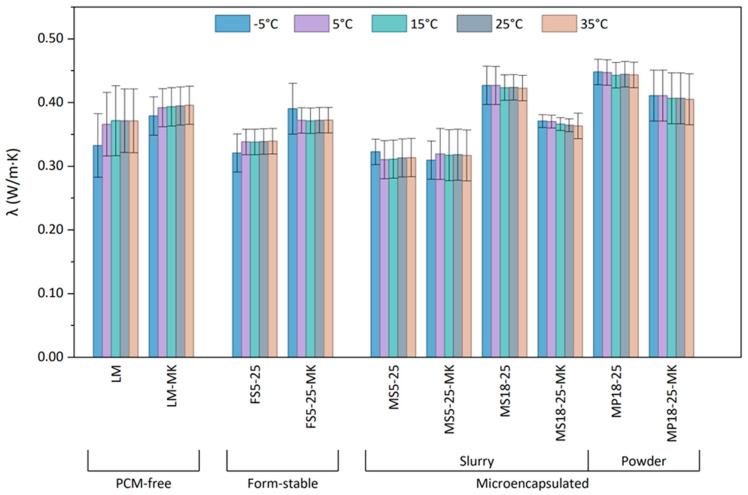
Thermal conductivity (λ) of multi-PCM lime mortars measured at −5, 5, 15, 25 and 35 °C.

The thermal performance of the developed mortars was first evaluated through thermal conductivity measurements within a representative operational temperature range (Figure 11). This approach allows characterization of heat transfer under conditions where the incorporated PCMs are in the solid state, undergoing phase transition, and fully melted, thus providing a comprehensive assessment under service-relevant conditions.

For the form-stable multi-PCM mortars, a comparison with the corresponding PCM-free reference mixtures reveals slightly lower thermal conductivity values (Figure 11). This behaviour can be attributed to the incorporation of phases with lower thermal conductivity than the mineral constituents of the lime matrix, such as paraffinic compounds and silica-based support, both known to exhibit low thermal conductivity [72,73,74,75,76]. Additionally, the broader and more heterogeneous pore structure identified by mercury intrusion porosimetry may further reduce heat transfer by disrupting conductive pathways within the matrix [77,78].

In mortars incorporating microencapsulated PCMs, thermal conductivity values remain close to those of the reference mixtures, with only minor variations observed. These differences are slightly more pronounced in specific formulations, such as MS5-25. Given that both the PCM core and encapsulating materials are similar across slurry- and powder-based systems, these variations are not attributed to compositional differences but rather to subtle microstructural changes, as indicated by the MIP results (Figure 4c–e).

The influence of temperature on thermal conductivity is relatively moderate across all formulations, with no abrupt variations within the investigated range. This indicates that, although phase change processes occur, their effect on steady-state thermal conductivity is limited. Consequently, the thermal contribution of the PCMs is primarily associated with latent heat storage rather than significant modifications of conductive heat transfer. This highlights the ability of the multi-PCM mortars to provide thermal regulation through energy storage while maintaining stable conductive properties under varying thermal conditions.

Overall, thermal conductivity is governed by both the intrinsic properties of the PCM phases and the resulting microstructure. All multi-PCM mortars exhibit values within the typical range for lime-based materials [61,62], indicating that multi-PCM incorporation does not significantly alter the conductive heat transfer.

Before analysing the phase-change behaviour of the PCM systems, their thermal stability was also examined to verify that the selected PCMs remain stable within the operational temperature range expected in building-envelope applications. Complementary TGA curves of the raw PCMs are provided in Supplementary Material Figures S4–S6 to compare the thermal stability and decomposition behaviour of the different PCM typologies. The form-stable PCMs showed a dominant mass-loss event between approximately 140 and 280 °C, associated with the volatilisation/decomposition of the paraffinic phase retained within the porous support [79,80]. After this event, both FS5 and FS25 showed a high final residue of around 40 wt.%, corresponding to the thermally stable silica carrier. In the slurry-microencapsulated PCMs, a slight mass loss around 100 °C was attributed to residual water from the aqueous medium. The main mass-loss process occurred at higher temperatures, approximately between 300 and 450 °C, and corresponded to the overlapping degradation of the paraffinic core and the melamine-formaldehyde shell [79,81]. Similarly, the powder-microencapsulated PCMs showed their main degradation process at high temperatures, approximately between 300 and 490 °C, associated with the combined decomposition of the paraffinic core and the melamine-based shell. The shift in the main degradation events towards higher temperatures in the microencapsulated systems indicates the protective role of the polymeric shell, which delays the thermal decomposition of the paraffinic core compared with the form-stable PCMs [79,80]. In both microencapsulated systems, the final residues were very low, confirming the predominantly organic nature of these PCMs. The TGA results therefore confirm that the selected PCM systems remain stable within the operational temperature range expected in building-envelope applications, supporting their suitability for use in thermally responsive lime mortars.

**Figure 12:**
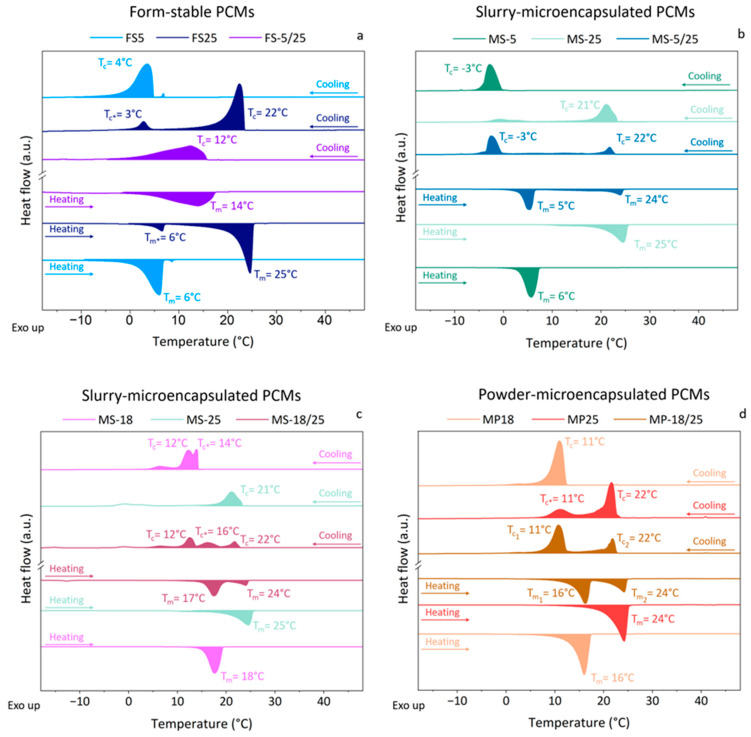
DSC thermal activation behaviour of single and multi-PCM systems with different PCM typologies: (**a**) form-stable PCMs (FS5, FS25 and FS-5/25), (**b**) slurry-microencapsulated PCMs (MS5, MS25 and MS-5/25), (**c**) slurry-microencapsulated PCMs (MS18, MS25 and MS-18/25), and (**d**) powder-microencapsulated PCMs (MP18, MP25 and MP-18/24). Melting (T_m_) and crystallization (T_c_) temperatures are indicated. * denotes a secondary peak attributed to supercooling phenomena, impurities, or solid–solid transitions, rather than the primary phase transition.

Beyond thermal conductivity and thermal stability, the thermal behaviour of mortars was further analysed through the thermal activation characteristics of the multi-PCM systems. Unlike single-PCM formulations, where the response is governed by a single-phase transition, multi-PCM systems involve multiple transitions, leading to a more complex behaviour. It is therefore essential to determine whether the combined system behaves as the sum of the individual PCM contributions or whether interactions between phases modify the overall thermal response. To address this, the melting and crystallization behaviour of both individual PCMs and their 1:1 mixtures, corresponding to the proportions used in the mortars, were analysed and the results are presented in Figure 12 and Table 5.

For the form-stable systems (Figure 12a), individual PCMs exhibit well-defined transitions. FS5 shows a melting peak at approximately 6 °C and a crystallization peak at 4 °C, with enthalpies of 68.9 J/g and 72.7 J/g, respectively. FS25 presents a melting peak of 25 °C and crystallization at 22 °C, with enthalpies of 87.8 J/g and 85.9 J/g (Table 5). Additionally, FS25 exhibits a secondary peak around 6 °C during melting and 3 °C during crystallization, likely associated with shorter chain paraffinic fractions [80,82] or solid–solid transitions commonly reported in n-alkanes [83,84,85].

When combined in a 1:1 ratio (FS-5/25), a markedly different thermal response is observed. Instead of two distinct phase transitions, the DSC curve shows a single broadened transition extending approximately from 0 to 18 °C (Figure 12a). The resulting peak is centred at around 14 °C during melting and 12 °C during crystallization (Table 5), with enthalpy values of 71.9 J/g and 72.4 J/g, respectively.

This deviation from additive behaviour indicates interactions between the paraffinic phases. In form-stable systems, the PCM is supported within a silica matrix but not fully confined, allowing contact between different alkane fractions. As a result, interactions, particularly in the liquid state, lead to a redistribution of the phase change over a broader temperature interval. Consequently, the system behaves as a single, integrated thermal storage phase with an extended activation range, rather than as two independent PCMs [86].

Focusing on the slurry-microencapsulated systems, Figure 12b (MS5-25) shows that the individual PCMs exhibit well-defined phase change transitions. MS5 displays a melting temperature of approximately 6 °C and a crystallization temperature of −3 °C, while MS25 shows melting and crystallization temperatures of around 25 °C and 21 °C, respectively. The corresponding melting enthalpies are 143.6 J/g for MS5 and 137.5 J/g for MS25, with crystallization enthalpies of 148.3 J/g and 141.1 J/g (Table 5). When combined in a 1:1 ratio, the DSC curve clearly shows two distinct phase change events, with peak positions remaining unchanged, indicating that each PCM retains its characteristic thermal behaviour.

A similar trend is observed in Figure 12c (MS18-25). The MS18 PCM exhibits a melting peak at approximately 18 °C, with an enthalpy of 160.9 J/g, and a crystallization process characterized by two closely spaced peaks at around 14 °C and 12 °C, with a total enthalpy of 159.6 J/g (Table 5). This double crystallization peak is likely associated with subcooling effects and multiple crystallization pathways within the paraffinic system [27,87]. In contrast, MS25 shows well-defined melting and crystallization transitions at approximately 25 °C and 21 °C, respectively.

When both PCMs are combined in a 1:1 ratio, the resulting DSC curve again displays two distinct phase change events corresponding to each PCM. The peak positions remain essentially unchanged (Figure 12c), indicating that the two PCMs behave independently. The crystallization region associated with MS18 appears slightly broadened in the mixture, reflecting the presence of its double-peak behaviour. Overall, the thermal response corresponds to the sum of the individual PCM contributions, with no evidence of strong interaction between phases.

In the case of the powder-microencapsulated system (Figure 12d, MP18-25), the individual PCMs exhibit melting peaks at approximately 16 °C and 24 °C, and crystallization peaks at around 11 °C and 22 °C for MP18 and MP25, respectively. The corresponding enthalpy values are 167.0 J/g and 181.0 J/g for melting, and 171.3 J/g and 181.0 J/g for crystallization (Table 5). A double peak is also observed during the crystallization of MP25, which may be attributed to supercooling effects [27].

When combined, the DSC curve shows two distinct transitions corresponding to each PCM, with no significant shift in peak position. This indicates that the system behaves as the sum of two independent phase change materials. This behaviour is consistently observed across all microencapsulated multi-PCM systems, regardless of whether the PCMs are incorporated in slurry or powder form. In all cases, the thermal response reflects the individual contributions of each PCM, with no clear evidence of interaction between phases. This behaviour is consistent with the microencapsulated nature of these systems, in which each paraffinic core is physically isolated by a polymeric shell. This shell-mediated confinement prevents direct contact between PCM domains, thereby preserving the independent phase change behaviour of each component. As a result, polymer-shell microencapsulation provides a more predictable route for designing multi-PCM systems with discrete and controllable thermal activation profiles.

**Figure 13:**
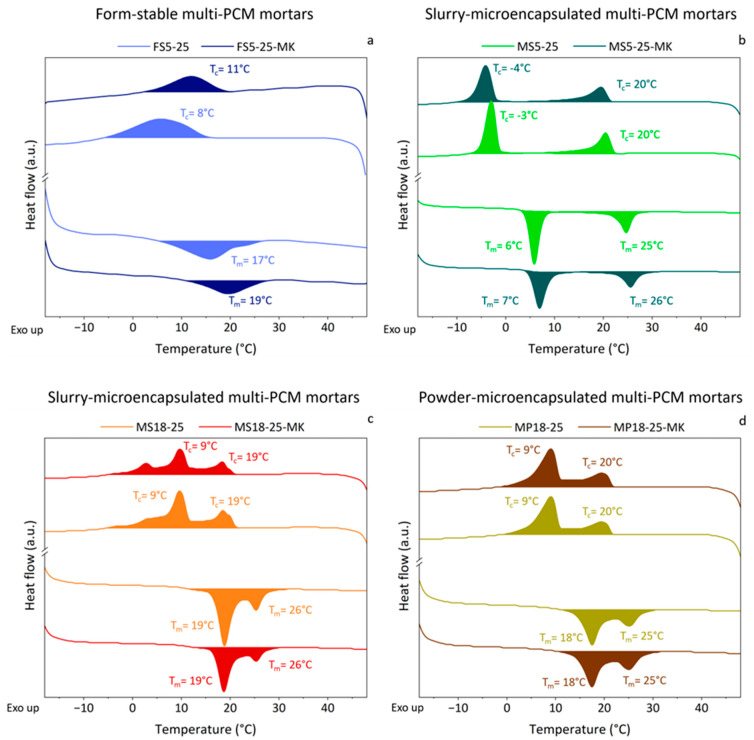
DSC thermal activation behaviour of multi-PCM mortars at 365 days of curing: (**a**) form-stable multi-PCM mortars (FS5-25 and FS5-25-MK), (**b**) slurry-microencapsulated multi-PCM mortars (MS5-25 and MS5-25-MK), (**c**) slurry-microencapsulated multi-PCM mortars (MS18-25 and MS18-25-MK) and (**d**) powder-microencapsulated multi-PCM mortars (MP18-25 and MP18-25-MK). Melting (T_m_) and crystallization (T_c_) temperatures are indicated.

Following the analysis of individual and combined PCM systems, the study was extended to the multi-PCM mortars to evaluate their effective thermal behaviour once incorporated into the lime matrix. In this context, the phase change behaviour of the mortars was analysed by DSC to quantify their heat storage and release capacity. The results are presented in Figure 13 and Table 6. The enthalpy values obtained for the multi-PCM mortars are consistent with those typically reported for PCM-containing mortars, reflecting the expected dilution effect within the mineral matrix [15,27].

For the form-stable mortars, the DSC response is characterized by a single, broad phase change transition (Figure 13a), consistent with the behaviour of the FS-5/25 system (Figure 12a). This transition is centred at approximately 17–19 °C during melting and shifts to 8–11 °C during crystallization (Table 6), indicating an extended activation range. The associated enthalpy values range between 1.0 and 1.4 J/g for melting and between 1.2 and 1.9 J/g for crystallization, confirming the effective contribution of the PCM phase within the composite.

For the slurry-microencapsulated MS5-25 mortars, the DSC response shows two distinct phase change transitions (Figure 13b), consistent with the corresponding PCM mixture (Figure 12b). The first transition occurs at approximately 6–7 °C during melting and −3 to −4 °C during crystallization, while the second is observed at around 25–26 °C during melting and 19–21 °C during crystallization (Table 6). The associated enthalpy values range from 2.0 to 2.3 J/g and 1.0 to 1.1 J/g for the two melting transitions, and from 2.2 to 2.5 J/g and 1.1 to 1.3 J/g for crystallization. The presence of two distinct transitions confirms that both PCM components remain thermally active within the mortar.

A similar behaviour is observed for the MS18-25 mortars (Figure 13c), where two distinct phase change transitions are also identified. The first transition occurs at approximately 18 °C during melting and around 9 °C during crystallization, while the second appears at approximately 25 °C during melting and 18 °C during crystallization (Table 6). The corresponding enthalpy values range between 1.3 and 1.7 J/g for the first melting transition and between 0.2 and 0.4 J/g for the second, with corresponding crystallization values between 1.3 and 1.7 J/g and 0.4 and 0.6 J/g, respectively.

A comparable response is obtained for the powder-microencapsulated MP18-25 mortars (Figure 13d), where two distinct phase change transitions are also clearly identified. The first transition occurs at approximately 18 °C during melting and around 9 °C during crystallization, while the second is centred at approximately 25 °C during melting and 20 °C during crystallization (Table 6). The associated enthalpy values are approximately 1.0 J/g and 0.3–0.4 J/g for the first and second melting transitions, respectively, with corresponding crystallization enthalpies of approximately 1.4–1.5 J/g and 0.4 J/g.

Overall, the thermal activation behaviour of the multi-PCM mortars closely reflects that observed for the corresponding PCM mixtures. Form-stable mortars exhibit a single, intermediate phase change transition, consistent with the FS-5/25 system, whereas microencapsulated mortars retain the distinct transitions of each PCM component, in line with the behaviour observed at the PCM level. This agreement indicates that incorporating PCMs into the lime matrix does not significantly modify their phase change behaviour. As a result, the thermal activation of multi-PCM mortars can be effectively designed based on the properties of the PCM mixtures, with their heat storage and release capacity preserved within the composite material.

From a practical design perspective, the DSC results provide useful guidance for selecting multi-PCM combinations according to the expected operating temperature range. The 5–25 °C systems provide the broadest activation window and are therefore better suited to colder or thermally variable building-envelope scenarios, where latent heat exchange over an extended temperature interval is required. In contrast, the 18–25 °C systems concentrate the phase-change response closer to comfort-related temperatures, making them more appropriate for temperate or mild-to-warm conditions in which thermal regulation is mainly required around indoor operating ranges. The PCM retention format also defines the type of thermal response obtained. Microencapsulated systems preserve more defined and additive phase-change contributions, reflecting the individual response of each PCM within the combined formulation. This makes them suitable when thermal activation needs to be targeted within specific temperature intervals. By contrast, form-stable systems generate broader and less discrete activation ranges, indicating that the combined response cannot be interpreted as a simple sum of the individual PCMs. This behaviour may be advantageous when a smoother and more extended thermal response is required across wider operating conditions.

The thermal performance of the multi-PCM lime mortars was further evaluated under dynamic conditions using a hotbox experimental setup, allowing assessment of their temperature buffering capacity at laboratory scale. A wide temperature cycling programme between −10 and 50 °C was applied to ensure complete phase transition of all incorporated PCMs within each formulation. This range covers the full activation window of both low- and high-transition PCMs, enabling evaluation of their combined thermal response under conditions where latent heat storage can be fully utilised.

**Figure 14:**
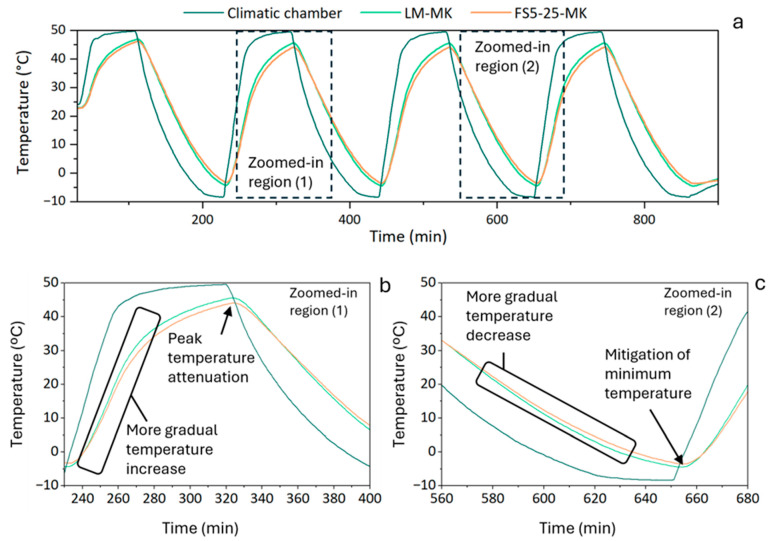
(**a**) Dynamic thermal response of the reference mortar (LM-MK) and multi-PCM mortar (FS5-25-MK) during hotbox cycling (−10 to 50 °C), showing temperature peak attenuation, smoother heating and cooling behaviour, and reduced minimum temperatures in the PCM-containing system; (**b**) Zoomed-in region 1 in heating stage; (**c**) Zoomed-in region 2 in cooling stage.

Such a temperature profile is also representative of environments with pronounced thermal fluctuations, including regions with high diurnal variations or strong seasonal thermal contrasts. The corresponding temperature evolution profiles are presented in Figure 14 for the reference mortar (LM-MK) and a representative PCM-containing formulation (FS5-25-MK). These profiles are representative of the behaviour observed across all multi-PCM mortars.

In general, PCM incorporation leads to a clear attenuation of temperature peaks and troughs, along with a more gradual heating and cooling response. This behaviour indicates increased thermal inertia associated with latent heat storage. In parallel, Table 7 summarises the quantitative thermal response, including differences in peak temperatures during heating and cooling stages, as well as the overall temperature deviation throughout the cycles. This provides a comprehensive comparison of the thermal performance of the different multi-PCM mortars.

As shown in Table 7, all multi-PCM formulations demonstrate a clear ability to reduce both peak temperatures during heating and minimum temperatures during cooling, with differences reaching up to 1.5 °C and 1.1 °C, respectively. These results confirm that the incorporation of multiple PCMs is effective in attenuating thermal extremes under dynamic conditions. This is particularly relevant given that the applied thermal programme ensures complete melting and crystallization of all PCMs, allowing each component to contribute to the overall thermal response.

Although some variations between formulations are observed, these cannot be directly linked to differences in PCM transition temperatures, as all PCMs undergo full phase change within the tested range. Instead, the observed differences are more likely related to variations in thermal conductivity and microstructural characteristics, which influence heat transfer and, consequently, the overall thermal behaviour of the mortars.

When the analysis is extended to the full heating and cooling stages, the magnitude of the thermal response becomes even more evident (Table 7). Maximum temperature differences reach up to 4.7 °C during heating for MP18-25-MK and up to 2.2 °C during cooling for MS18-25. These results confirm that the thermal buffering effect is not limited to peak conditions but is sustained throughout the entire thermal cycle. This behaviour is consistent with the temperature evolution shown in Figure 14, where the PCM-containing mortars exhibit more gradual heating and cooling compared to the PCM-free reference, reflecting the increase in thermal inertia associated with multi-PCM incorporation. In this context, the effect of PCMs is expressed not only through the attenuation of maximum and minimum temperatures, but also through a smoother thermal response during both heating and cooling stages, contributing to improved thermal stability and comfort.

Furthermore, integrating the temperature difference over time provides an estimate of the relative energy exchange, with values reaching up to 8.2·10^5^ °C·s/m^2^ for MS18-25. The consistently high values obtained across all formulations indicate that the thermal contribution of the PCMs is maintained throughout the entire cycle, reinforcing the ability of the mortars to dampen temperature fluctuations under dynamic conditions. This sustained response is directly associated with increased thermal inertia and highlights the potential of these systems to enhance thermal regulation and energy efficiency.

Importantly, both the temperature differences and the integrated thermal response fall within the range reported in the literature for lime mortars incorporating single-PCMs under comparable thermal cycling conditions [15,45,88], confirming that the use of multi-PCM systems preserves the expected level of thermal performance while extending its effectiveness over a wider temperature interval, as supported by the DSC analyses (Figure 12 and Figure 13).

Overall, these findings highlight the multi-PCM approach as a promising strategy for developing thermally responsive lime mortars under variable operating conditions. By distributing the thermal effect across multiple phase transition intervals, rather than concentrating it around a single temperature, multi-PCM systems enable a broader and more continuous thermal response. This behaviour is particularly advantageous under dynamic conditions and provides a flexible framework for designing mortar formulations tailored to specific climatic profiles.

### 3.6. Sustainability and Cost Assessment

Following the demonstrated improvements in thermal performance and energy efficiency, the environmental impact of the developed mortars was evaluated through their global warming potential (GWP) within the A1–A3 stages. The GWP of individual raw materials was obtained from the Ecoinvent v3.9.1 database or from manufacturer-provided data, depending on availability (Table 8). For PCM systems, in the absence of environmental product declarations, their GWP was estimated based on their composition, considering the mass contribution of each constituent material (Table 8). This approach ensures a consistent and comparable evaluation across all formulations.

Table 8 indicates that silica-supported PCMs exhibit lower GWP values than microencapsulated systems. This difference is primarily attributed to their composition, particularly the presence of polymeric shells, which are associated with higher environmental impacts. In addition, the production of form-stable PCMs is generally less complex and less energy-intensive than microencapsulation processes [89,90,91], further contributing to their lower GWP.

The transport stage (A2) was evaluated considering the origin of each raw material and typical transport routes to Pamplona (Spain), using an emission factor of 0.0912 kg CO_2_-eq per tonne·km, corresponding to a 33-tonne articulated lorry. The manufacturing stage (A3) was calculated using a factor of 0.0007 kg CO_2_-eq per kg of fresh mortar, representative of wet batching processes. Both values were obtained from the ICE Database v1.2 (Inventory of Carbon and Energy, December 2024) and applied consistently across all formulations.

**Figure 15:**
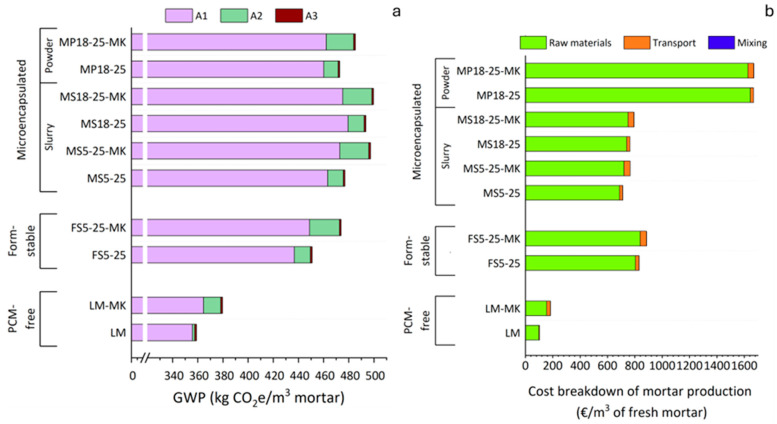
Cradle-to-gate environmental and economic assessment of lime-based mortars incorporating PCM systems: (**a**) GWP (A1–A3) and (**b**) cost breakdown of raw materials, transport and mixing.

The overall A1–A3 results (Figure 15a) show that PCM incorporation increases GWP compared to reference mortars, with total increments of approximately 25–35%, primarily driven by PCM composition. The A1 stage dominates the total impact. Among the systems, form-stable mortars (FS5-25) exhibit the lowest GWP (≈436.7 kg CO_2_e/m^3^), followed by powder microencapsulated systems (MP18-25) (≈460.1 kg CO_2_e/m^3^), while slurry systems (MS5-25 and MS18-25) show the highest values (≈463.2 and 479.3 kg CO_2_e/m^3^, respectively).

A similar trend is observed for MK-containing mortars, which exhibit slightly higher GWP due to the additional contribution of metakaolin. However, this increase should be considered alongside the improved durability observed for MK-bearing mortars (Figure 9), which may extend service life and lead to a more favourable overall environmental balance [4,92,93]. The contribution of transport (A2) remains relatively small, ranging from approximately 11 and 23 kg CO_2_e/m^3^, while the manufacturing stage (A3) contributes around 1.3 kg CO_2_e/m^3^ across all formulations, confirming its negligible influence on total GWP (Figure 15a).

Overall, although PCM incorporation increases the embodied GWP of the mortars, this increase remains moderate and is inherit to the addition of a functional component (Figure 15a). Importantly, PCM composition plays a key role in controlling this impact, with silica-supported systems offering a clear environmental advantage. When considered alongside the improvements in thermal performance, the use of appropriately selected PCM systems enables a balanced approach in which gains in operational energy efficiency can offset the additional embodied impact, supporting the development of more sustainable lime-based mortars [4].

In addition to the environmental assessment, the economic performance of the developed mortars was analysed in order to provide a more comprehensive evaluation of their feasibility (Figure 15b). The aim of this analysis is to compare production costs as a function of PCM typology and format. Within the cradle-to-gate scope considered (A1–A3), an increase in cost for PCM-containing mortars relative to PCM-free systems is expected, as it reflects the incorporation of an additional functional component. Nevertheless, this stage offers a useful basis for preliminary screening of PCM solutions, enabling comparative evaluation and supporting material design decisions.

Consistent with the GWP results (Figure 15a), the cost assessment (Figure 15b) shows that the total production cost is primarily governed by raw materials, while transport and mixing remain negligible. As expected, PCM incorporation leads to a significant increase in cost, with values rising from approximately 103.3 €/m^3^ for the reference mortar (LM) to as high as 1669.6 €/m^3^ depending on PCM type.

Clear differences are observed between PCM systems (Figure 15b). Slurry-based microencapsulated formulations (MS5-25 and MS18-25) present the lowest costs among PCM-containing mortars, ranging between approximately 711.6 and 794.3 €/m^3^. Form-stable systems (FS5-25) show intermediate values ca. 830.7–885.8 €/m^3^, representing an increase of around 13% compared to slurry systems. In contrast, powder-based microencapsulated systems (MP18-25) exhibit substantially higher costs, reaching up to 1669.6 €/m^3^, approximately 120% higher than slurry formulations. These differences are mainly driven by the cost of PCM itself, which dominates the overall economic behaviour of the mortars. From this perspective, slurry-based systems emerge as the most cost-efficient option, while powder microencapsulated systems are considerably less competitive. Form-stable systems represent an intermediate solution, offering a balance between cost and environmental performance.

It is important to recognise that PCM-containing mortars inherently involve higher initial costs and embodied impacts due to the addition of a functional phase. However, these increases should not be assessed in isolation but rather within a life cycle perspective. The present analysis is limited to cradle-to-gate stages (A1–A3), and therefore does not account for potential benefits during the use phase. Extensive literature and real-scale applications demonstrate that improved thermal performance, particularly when PCM transition temperatures are well matched to climatic conditions, can lead to significant reductions in operational energy demand [4,12,13,15,27,88,94]. In addition, the enhanced durability observed in MK-containing mortars may extend service life and reduce maintenance and replacement needs over time [4,92,93]. Although these aspects are not quantified in this study, they are expected to play a key role in the overall life cycle performance. From this perspective, the initial increase in cost and embodied impact may be progressively offset during the service life of the material.

Furthermore, the continued development and wider adoption of PCM technologies are likely to improve market competitiveness and reduce costs over time, strengthening their economic viability. This is particularly relevant under current and future climate scenarios, characterized by increasing thermal extremes, where improving energy efficiency remains a central objective, as reflected in European decarbonisation strategies [37,95,96,97].

Overall, these results highlight that PCM selection is a critical design parameter, as it directly influences both environmental and economic performance and ultimately determines the feasibility of multi-PCM lime mortars from a life cycle perspective.

## 4. Conclusions

Multi-PCM lime mortars were successfully developed by combining PCMs with low and high transition temperatures (5–25 °C), as well as intermediate and high temperatures (18–25 °C), incorporating both form-stable systems and microencapsulated PCMs in slurry and powder formats. All formulations were optimised using polymeric chemical additives to include 20% PCM (10% + 10% bwol), achieving cohesive, crack-free renders with adequate consistency and full adhesion, confirming their suitability for building envelope applications.

From a microstructural perspective, PCM typology plays a key role in defining the internal structure of the mortars. Form-stable PCMs resulted in more heterogeneous pore networks, whereas microencapsulated systems preserved pore structures similar to PCM-free mortars. Metakaolin incorporation led to moderate pore refinement, mainly attributed to its filler effect and limited pozzolanic activity under the studied conditions.

In terms of mechanical performance, compressive strength was generally maintained across all multi-PCM formulations. Form-stable systems exhibited the highest values (up to ca. 3 MPa), while powder microencapsulated mortars showed the lowest (ca. 1 MPa). The addition of metakaolin produced moderate strength improvements, consistent with the observed microstructural refinement.

Durability results highlighted the importance of matrix composition. All formulations showed good resistance to salt crystallization, completing 28 cycles with limited damage. Under freeze–thaw conditions, metakaolin-containing mortars demonstrated improved performance, in some cases outperforming PCM-free references.

Thermal conductivity remained within the typical range for lime mortars, indicating that PCM incorporation does not significantly affect conductive heat transfer when formulations are properly optimized.

Thermal activation, evaluated by differential scanning calorimetry (DSC), revealed distinct behaviours depending on PCM typology. Form-stable systems exhibited a single broadened phase transition due to interactions between paraffinic components, whereas polymeric microencapsulated systems preserved the individual transitions of each PCM. In both cases, the use of multiple PCMs effectively expanded the thermal activation range. This behaviour was maintained at the mortar scale, confirming that the lime matrix does not alter the intrinsic phase change properties of the PCMs.

Under dynamic conditions, hotbox testing demonstrated a sustained temperature buffering effect across all multi-PCM mortars. Peak reductions of up to 1.5 °C (heating) and 1.1 °C (cooling) were observed, with maximum differences reaching 4.7 °C and 2.2 °C, respectively. The integrated thermal response reached up to 8.2 × 10^5^ °C·s/m^2^, confirming continuous heat absorption and release and highlighting the strong potential of these materials for improving thermal regulation and indoor comfort.

From an environmental perspective, PCM incorporation increased cradle-to-gate impacts (A1–A3) by approximately 25–35%, mainly due to raw material production (A1), particularly paraffinic cores and encapsulation materials. Form-stable systems exhibited the lowest impacts, while microencapsulated systems, especially in powder form, showed the highest. Transport (A2) and mixing (A3) contributions remained minor.

A similar trend was observed in the economic assessment, with PCM-containing mortars presenting higher initial costs. Slurry-based microencapsulated systems were the most cost-effective, while powder systems were significantly more expensive. However, these results should be interpreted within a life cycle perspective. Improved thermal performance and enhanced durability are expected to reduce operational energy demand and extend service life, potentially offsetting the initial environmental and economic costs.

Despite the comprehensive multi-scale characterization performed in this work, the present study should be understood as a robust foundational step towards the development of optimized multi-PCM lime mortars rather than as a definitive full-scale implementation assessment. The experimental programme successfully demonstrates the technical feasibility, mechanical viability, thermal functionality, durability potential, and preliminary sustainability implications of these systems under controlled laboratory conditions, thereby establishing a solid scientific basis for their future application. Nevertheless, certain aspects warrant further investigation to fully consolidate their large-scale applicability. In particular, the environmental and economic analyses were limited to cradle-to-gate stages (A1–A3), meaning that the broader life cycle benefits associated with operational energy savings and service-life extension remain to be fully quantified. Likewise, while hotbox testing provided valuable dynamic thermal performance insights, further real-scale building simulations and long-term in situ studies under diverse climatic conditions would strengthen the quantification of their practical energy-saving potential. In addition, although the durability programme included highly aggressive freeze–thaw and salt attack regimes, future work should extend the assessment towards longer-term service-related ageing, thermal cycling and application-scale monitoring under representative exposure conditions. In this regard, application-scale validation is already being advanced within an ongoing research framework focused on PCM-enhanced renders for building envelope retrofitting. In this parallel work, single-PCM render mortars have been applied in pilot-scale façade mock-ups and monitored under outdoor exposure, providing an initial step towards the assessment of PCM-bearing lime-based renders under real environmental boundary conditions. These preliminary application-scale results have shown that PCM incorporation can smooth temperature fluctuations and improve the thermal behaviour of façade systems. Building upon this first validation stage, the multi-PCM formulations developed in the present study provide a scientific basis for future façade-scale testing, where their performance can be evaluated and compared with that of single-PCM systems under representative exposure conditions. Future research should therefore build upon the strong framework established in this work by extending towards full life cycle and building-scale evaluations, while also exploring further optimization of PCM combinations, encapsulation strategies, and lower-impact PCM alternatives. Such developments will further advance multi-PCM lime mortars from highly promising laboratory-validated systems towards fully implemented sustainable and climate-adaptive construction solutions.

Overall, the multi-PCM approach effectively expands the thermal operating range of lime mortars, enabling improved thermal regulation without compromising mechanical performance or durability. These findings provide a strong basis for the development of tailored, climate-responsive building materials. The total cost of each formulation was calculated according to its composition, following an industrial-scale perspective representative of real construction scenarios. This approach ensures consistency between environmental and economic evaluations, providing a comparable framework for assessing both embodied carbon and cost at the material level.

## Figures and Tables

**Table 1:** Multi-PCM mortar formulations: PCM typology, specifications, and thermal activation temperatures.

PCM Typology	Mixture	Format	Retention Mechanism	Melting Temperatures PCM_1_/PCM_2_ (°C)	PCM_1_/PCM_2_ (% bwol)
Form-stable	FS5-25	Powder	Silica-supported	5/25	10/10
	FS5-25-MK				
Micro-encapsulated	MS5-25	Slurry	Encapsulation within a melamine-formaldehyde shell	5/25	10/10
	MS5-25-MK				
	MS18-25			18/25	10/10
	MS18-25-MK				
	MP18-25	Powder	Encapsulation within a melamine shell	18/25	10/10
	MP18-25-MK				

**Table 2:** Mix design of reference and multi-PCM lime mortars.

Mixture	Air Lime (g)	Calcitic Sand (g)	MK (g)	PCM_1_ (g)	PCM_2_ (g)	SP (g)	Adhesion Booster (g)	Water/ Total Solids
LM	217.0	783.0	-	-	-	1.3	1.1	0.25
LM-MK	217.0	783.0	43.4	-	-	1.6	1.1	0.25
FS5-25	217.0	783.0	-	21.7	21.7	2.2	1.1	0.25
FS5-25-MK	217.0	783.0	43.4	21.7	21.7	2.2	1.1	0.25
MS5-25	217.0	783.0	-	21.7	21.7	1.9	1.1	0.25
MS5-25-MK	217.0	783.0	43.4	21.7	21.7	1.9	1.1	0.25
MS18-25	217.0	783.0	-	21.7	21.7	1.9	1.1	0.25
MS18-25-MK	217.0	783.0	43.4	21.7	21.7	1.8	1.1	0.25
MP18-25	217.0	783.0	-	21.7	21.7	1.6	1.1	0.25
MP18-25-MK	217.0	783.0	43.4	21.7	21.7	1.6	1.1	0.25

**Table 3:** Damage classification system used for visual evaluation of specimens subjected to durability cycles.

Damage Scale (0–10)	Characteristics
0	No visible damage; specimen intact.
1	Minimal material loss; almost no damage.
2	Small cracks; slight material loss.
3	Moderate cracks; visible wear.
4	Larger cracks; significant material loss.
5	Major cracks; pronounced material loss.
6	Severe cracks; surface starting to break apart.
7	Large sections missing; extensive cracking.
8	Structure heavily compromised; crumbling.
9	Near total destruction; disintegration visible.
10	Total destruction; specimen turned to dust.

**Table 4:** Fresh-state properties and qualitative rendering performance of optimised lime-based mortars incorporating different PCM systems.

Mixture	Consistency (mm)	Fresh Density (kg/L)	Entrapped Air (%)	Water Retentivity (%)	Rendering Evaluation (0–3)
LM	182	1.945	4.3	95.9	3
LM-MK	175	1.943	2.4	95.6	3
FS5-25	180	1.866	2.5	94.5	3
FS5-25-MK	170	1.901	2.0	95.2	3
MS5-25	205	1.820	3.8	93.3	3
MS5-25-MK	185	1.849	3.6	94.0	2
MS18-25	185	1.883	2.7	94.9	3
MS18-25-MK	189	1.858	4.4	96.5	3
MP18-25	195	1.866	3.2	94.0	3
MP18-25-MK	163	1.862	3.2	94.0	2

**Table 5:** DSC analysis of single and multi-PCM systems: phase transition temperatures, enthalpies, and thermal profiles.

Single-PCM	PCM Type	ΔH_m_ (J/g)	T_m_ (°C)	ΔH_c_ (J/g)	T_c_ (°C)
FS5	Form-stable	68.9 ± 0.2	6.0 ± 0.1	72.7 ± 0.1	3.5 ± 0.1
FS25	Form-stable	87.8 ± 0.1	24.6 ± 0.1	85.9 ± 0.1	22.4 ± 0.1
MS5	Slurry- microencapsulated	143.6 ± 0.3	5.7 ± 0.1	148.3 ± 0.2	−2.9 ± 0.1
MS25	Slurry- microencapsulated	137.5 ± 0.6	24.5 ± 0.1	141.1 ± 0.1	21.0 ± 0.1
MS18	Slurry- microencapsulated	160.9 ± 0.6	17.6 ± 0.1	159.6 ± 0.6	12.1 ± 0.1
MP18	Powder- microencapsulated	167.0 ± 0.1	16.0 ± 0.1	171.3 ± 0.1	10.9 ± 0.1
MP25	Powder- microencapsulated	167.9 ± 0.1	24.2 ± 0.1	181.0 ± 0.1	21.6 ± 0.1
**Multi-PCM**	**Phase Transition Profile**	**ΔH_m_ (J/g)**	**T_m_ (°C)**	**ΔH_c_ (J/g)**	**T_c_ (°C)**
FS-5/25	Single peak	71.9 ± 0.3	13.8 ± 0.1	72.4 ± 0.2	12.3 ± 0.1
MS-5/25	Multiple peaks:				
	Peak_1_	81.3 ± 0.3	5.4 ± 0.1	80.3 ± 0.6	−2.5 ± 0.1
	Peak_2_	42.0 ± 0.8	23.9 ± 0.1	37.9 ± 0.6	21.7 ± 0.1
MS-18/25	Multiple peaks:				
	Peak_1_	84.4 ± 0.3	17.4 ± 0.1	80.2 ± 0.1	12.5 ± 0.1
	Peak_2_	20.4 ± 0.1	24.0 ± 0.1	21.1 ± 0.2	21.7 ± 0.1
MP-18/25	Multiple peaks:				
	Peak_1_	86.5 ± 0.5	16.2 ± 0.1	101.7 ± 0.4	10.7 ± 0.1
	Peak_2_	45.4 ± 0.4	24.3 ± 0.3	37.7 ± 0.2	21.8 ± 0.1

**Table 6:** DSC analysis of multi-PCM mortars: transition temperatures, enthalpies and phase transition profiles.

Mixture	Phase Transition Profile	ΔH_m_ (J/g)	T_m_ (°C)	ΔH_c_ (J/g)	T_c_ (°C)
FS5-25	Single peak	1.4 ± 0.1	17.4 ± 0.1	1.9 ± 0.1	8.2 ± 0.1
FS5-25-MK	Single peak	1.0 ± 0.1	19.1 ± 0.1	1.2 ± 0.1	11.2 ± 0.1
MS5-25	Multiple peaks:				
	Peak_1_	2.3 ± 0.1	5.9 ± 0.1	2.5 ± 0.1	−3.0 ± 0.1
	Peak_2_	1.1 ± 0.1	24.7 ± 0.1	1.3 ± 0.1	20.5 ± 0.1
MS5-25-MK	Multiple peaks:				
	Peak_1_	2.0 ± 0.1	6.9 ± 0.1	2.2 ± 0.1	−4.2 ± 0.1
	Peak_2_	1.0 ± 0.1	25.6 ± 0.1	1.1 ± 0.1	19.6 ± 0.1
MS18-25	Multiple peaks:				
	Peak_1_	1.7 ± 0.1	18.8 ± 0.1	1.7 ± 0.1	9.4 ± 0.1
	Peak_2_	0.4 ± 0.1	25.5 ± 0.1	0.6 ± 0.1	18.7 ± 0.1
MS18-25-MK	Multiple peaks:				
	Peak_1_	1.3 ± 0.1	18.7 ± 0.1	1.3 ± 0.1	9.5 ± 0.1
	Peak_2_	0.2 ± 0.1	25.5 ± 0.1	0.4 ± 0.1	18.6 ± 0.1
MP18-25	Multiple peaks:				
	Peak_1_	1.0 ± 0.1	17.5 ± 0.1	1.5 ± 0.1	9.0 ± 0.1
	Peak_2_	0.3 ± 0.1	25.4 ± 0.1	0.4 ± 0.1	20.1 ± 0.1
MP18-25-MK	Multiple peaks:				
	Peak_1_	1.0 ± 0.1	17.5 ± 0.1	1.4 ± 0.1	9.0 ± 0.1
	Peak_2_	0.4 ± 0.1	25.3 ± 0.1	0.4 ± 0.1	20.2 ± 0.1

**Table 7:** ΔT at maximum and minimum peak temperatures, maximum ΔT during heating and cooling phases, and total relative energy exchanged between multi-PCM and reference hotboxes (−10 to 50 °C). Temperature differences (ΔT) are calculated as the absolute value of the difference between the PCM-free hotbox and the multi-PCM hotbox (ΔT=$\;\left|T_{PCM-free\;hotbox}-T_{Multi-PCM\;hotbox}\right|$).

	|ΔT| at Peak Temperatures (°C)		Maximum |ΔT| During Stages (°C)		Relative Energy Exchanged (°C⋅s/m^2^)
Mixture	At T_max_	At T_min_	Heating	Cooling	Total
FS5-25	0.8 ± 0.1	0.2 ± 0.1	2.5 ± 0.1	0.5 ± 0.1	5.0·10^5^
FS5-25-MK	1.5 ± 0.1	1.0 ± 0.1	2.6 ± 0.1	1.6 ± 0.1	7.7·10^5^
MS5-25	0.8 ± 0.3	0.6 ± 0.1	2.3 ± 0.1	0.7 ± 0.1	5.1·10^5^
MS5-25-MK	0.5 ± 0.1	0.8 ± 0.1	3.7 ± 0.7	1.1 ± 0.1	6.4·10^5^
MS18-25	0.8 ± 0.2	1.1 ± 0.1	3.8 ± 0.4	2.2 ± 0.1	8.2·10^5^
MS18-25-MK	0.9 ± 0.2	0.2 ± 0.1	3.5 ± 0.1	1.4 ± 0.1	6.4·10^5^
MP18-25	1.1 ± 0.2	0.7 ± 0.1	3.2 ± 0.1	1.8 ± 0.1	7.5·10^5^
MP18-25-MK	0.5 ± 0.1	0.4 ± 0.1	4.7 ± 0.7	1.5 ± 0.1	7.0·10^5^

**Table 8:** Cradle-to-gate GWP of raw materials and PCM systems adopted in the multi-PCM lime mortars, including data sources and composition-based estimates.

Material	Source	GWP (kg CO_2_e/kg)
Hydrated air lime	European Lime Association EPD	0.874
Calcitic sand	EPD-S-P 11716	0.00273
Metakaolin	EPD-S-P 03756	0.35937
Superplasticizer	EPD-EFC 20210198-IBG1-EN	1.53
Adhesion booster	Manufacturer data	32.6
Paraffin wax	Ecoinvent v3.9.1	1.5
Silica (activated)	Ecoinvent v3.9.1	2.11
Melamine-formaldehyde	Ecoinvent v3.9.1	5.84
Melamine	Ecoinvent v3.9.1	4.69
PCM systems:		
FS-PCM	60% paraffinic core + 40% porous silica	1.744
MS-PCM	80% paraffinic core + 20% melamine-formaldehyde	2.368
MP-PCM	80% paraffinic core + 20% melamine shell	2.138

## Data Availability

Data will be available upon request.

## Supplementary Materials

The following supporting information can be downloaded at https://www.mdpi.com/article/10.3390/polym18121481/s1. Figure S1. SEM micrograph and EDS elemental maps of the pure form-stable PCM FS5: (a) SEM micrograph, (b) Si elemental map, (c) O elemental map and (d) C elemental map. Figure S2. SEM micrograph and EDS elemental maps of the pure slurry-microencapsulated PCM MS18: (a) SEM micrograph, (b) C elemental map and (c) N elemental map. Figure S3. SEM micrograph and EDS elemental map of the pure powder-microencapsulated PCM MP18: (a) SEM micrograph and (b) C elemental map. Figure S4. TGA curves of the pure form-stable PCMs: FS25 and FS5. Figure S5. TGA curves of the pure slurry-microencapsulated PCMs: MS5, MS18 and MS25. Figure S6. TGA curves of the pure powder-microencapsulated PCMs: MP18 and MP25.

## References

[1] Zhai X.R., Xu Z.Y., Zhang W., Zhang Q.Q., Yang X.F., Qu J.Z., Liu G.L., Yu B. (2025). Phase change thermal energy storage: Materials and heat transfer enhancement methods. J. Energy Storage.

[2] Masood U., Haggag M., Hassan A., Laghari M. (2023). A Review of Phase Change Materials as a Heat Storage Medium for Cooling Applications in the Built Environment. Buildings.

[3] Podara C.V., Kartsonakis I.A., Charitidis C.A. (2021). Towards phase change materials for thermal energy storage: Classification, improvements and applications in the building sector. Appl. Sci..

[4] Rubio-Aguinaga A., Kyriakou L., Fernández J.M., Navarro-Blasco I., Pavia S., Álvarez J.I. (2025). Sustainability of PCM-lime mortars for heritage retrofitting: Carbon footprint and impact on energy demand across climates. Case Stud. Constr. Mater..

[5] Jouhara H., Żabnieńska-Góra A., Khordehgah N., Ahmad D., Lipinski T. (2020). Latent thermal energy storage technologies and applications: A review. Int. J. Thermofluids.

[6] Elenga R.G., Zhu L., Defilla S. (2025). Performance evaluation of different building envelopes integrated with phase change materials in tropical climates. Energy Built Environ..

[7] Li Q., Ju Z.P., Wang Z.G., Ma L.Y., Jiang W., Li D., Jia J.J. (2022). Thermal performance and economy of PCM foamed cement walls for buildings in different climate zones. Energy Build..

[8] Staszczuk A., Kuczynski T. (2021). The impact of wall and roof material on the summer thermal performance of building in a temperate climate. Energy.

[9] Liu L.S., Hammami N., Trovalet L., Bigot D., Habas J.P., Malet-Damour B. (2022). Description of phase change materials (PCMs) used in buildings under various climates: A review. J. Energy Storage.

[10] Akeiber H., Nejat P., Majid M.Z.A., Wahid M.A., Jomehzadeh F., Famileh I.Z., Calautit J.K., Hughes B., Zaki S.A. (2016). A review on phase change material (PCM) for sustainable passive cooling in building envelopes. Renew. Sustain. Energy Rev..

[11] Osibodu S.J., Adeyinka A.M., Mbelu O.V. (2024). Phase change material integration in concrete for thermal energy storage: Techniques and applications in sustainable building. Sustain. Energy Res..

[12] Saffari M., de Gracia A., Fernández C., Cabeza L.F. (2017). Simulation-based optimization of PCM melting temperature to improve the energy performance in buildings. Appl. Energy.

[13] Al Jebaei H., Aryal A., Jeon I.K., Azzam A., Kim Y.R., Baltazar J.C. (2024). Evaluating the potential of optimized PCM-wallboards for reducing energy consumption and CO_2_ emission in buildings. Energy Build..

[14] Wadee A., Walker P., Mccullen N., Ferrandiz-Mas V. (2025). The effect of thermal cycling on the thermal and chemical stability of paraffin phase change materials (PCMs) composites. Mater. Struct..

[15] Rubio-Aguinaga A., Kyriakou L., Fernández J.M., Navarro-Blasco Í., Álvarez J.I. (2025). Microstructural analysis of bio-based PCM-enhanced lime mortars: Durability and energy efficiency for sustainable buildings. Constr. Build. Mater..

[16] Sarcinella A., De Aguiar J.L.B., Lettieri M., Cunha S., Frigione M. (2020). Thermal Performance of Mortars Based on Different Binders and Containing a Novel Sustainable Phase Change Material (PCM). Materials.

[17] Rahemipoor S., Bayat M., Hasany M., Mehrali M., Almdal K., Ranjbar N., Mehrali M. (2024). Microencapsulated phase change material in 3D-printable mortars. Energy Convers. Manag..

[18] Cunha S., Castro J., Aguiar J.B. (2023). Impact of gypsum mortars functionalized with phase change materials in buildings. J. Energy Storage.

[19] Theodoridou M., Kyriakou L., Ioannou I. (2026). Optimized lime-based renders with Phase Changing Materials (PCMs) for energy-efficient and climate resilient traditional and contemporary structures. Energy Build..

[20] Kishore R.A., Booten C., Bianchi M.V.A., Vidal J., Jackson R. (2022). Evaluating cascaded and tunable phase change materials for enhanced thermal energy storage utilization and effectiveness in building envelopes. Energy Build..

[21] Ha J., Choi Y., Lee S., Oh K. (2020). Diurnal and Seasonal Variations in the Effect of Urban Environmental Factors on Air Temperature: A Consecutive Regression Analysis Approach. Int. J. Environ. Res. Public Health.

[22] Shi Z.P., Yang J., Wang L.E., Lv F., Wang G.Y., Xiao X.M., Xia J.H. (2022). Exploring seasonal diurnal surface temperature variation in cities based on ECOSTRESS data: A local climate zone perspective. Front. Public Health.

[23] Liu H., Wu Y.X., Li B.Z., Cheng Y., Yao R.M. (2017). Seasonal variation of thermal sensations in residential buildings in the Hot Summer and Cold Winter zone of China. Energy Build..

[24] D’Agostino D., Congedo P.M., Baglivo C., Albanese P.M., Colazzo B., Colazzo L. (2025). Assessing the Impact of Climate Change on Energy Demand in European Existing Buildings and Future NZEBs.

[25] Panchal J.M., Modi K.V., Patel V.J. (2022). Development in multiple-phase change materials cascaded low-grade thermal energy storage applications: A review. Clean. Eng. Technol..

[26] Narasimhan N.L. (2019). Assessment of latent heat thermal storage systems operating with multiple phase change materials. J. Energy Storage.

[27] Rubio-Aguinaga A., Fernández J.M., Navarro-Blasco Í., Álvarez J.I. (2024). Air lime renders with microencapsulated phase change materials: Assessment of microstructural and thermal properties. Constr. Build. Mater..

[28] Rubio-Aguinaga A., Fernandez J.M., Navarro-Blasco I., Alvarez J.I. (2024). Study on the Interaction of Polymeric Chemical Additives with Phase Change Materials in Air Lime Renders. Polymers.

[29] Ju J.T., Cao H.B., Guo W.K., Luo N., Zhang Q.M., Wang Y.G. (2024). Experimental Study on Calcination of Portland Cement Clinker Using Different Contents of Stainless Steel Slag. Materials.

[30] Ryan J., Bussmann M., DeMartini N. (2022). CFD Modelling of Calcination in a Rotary Lime Kiln. Processes.

[31] Manoharan A., Umarani C. (2022). Lime Mortar, a Boon to the Environment: Characterization Case Study and Overview. Sustainability.

[32] Van Balen K. (2005). Carbonation reaction of lime, kinetics at ambient temperature. Cem. Concr. Res..

[33] Rodriguez-Navarro C., Ilic T., Ruiz-Agudo E., Elert K. (2023). Carbonation mechanisms and kinetics of lime-based binders: An overview. Cem. Concr. Res..

[34] Veiga M.D., Fragata A., Velosa A.L., Magalhaes A.C., Margalha G. (2010). Lime-Based Mortars: Viability for Use as Substitution Renders in Historical Buildings. Int. J. Archit. Herit..

[35] Silva B.A., Guerreiro E., Duarte A.P.C. (2025). Comparative study of lime-based mortars for conservation and restoration interventions. Constr. Build. Mater..

[36] Pavlíková M., Zemanová L., Záleská M., Pokorny J., Lojka M., Jankovsky O., Pavlík Z. (2019). Ternary Blended Binder for Production of a Novel Type of Lightweight Repair Mortar. Materials.

[37] Commission E. (2020). A Renovation Wave for Europe—Greening Our Buildings, Creating Jobs, Improving Lives.

[38] Lucchi E. (2022). Energy Efficiency of Historic Buildings. Buildings.

[39] (2006). Methods of Test for Mortar for Masonry. Part 3: Determination of Consistence of Fresh Mortar (by Flow Table).

[40] (1999). Methods of Test for Mortar for Masonry—Part 6: Determination of Bulk Density of Fresh Mortar.

[41] (1999). Methods of Test for Mortar for Masonry. Part 7: Determination of Air Content of Fresh Mortar.

[42] (1993). Test Methods. Fresh Mortars. Determination of Water Retentivity.

[43] (2008). Tests for Thermal and Weathering Properties of Aggregates—Part 1: Determination of Resistance to Freezing and Thawing.

[44] (2009). Tests for Thermal and Weathering Properties of Aggregates—Part 2: Magnesium Sulfate Test.

[45] Rubio-Aguinaga A., Kyriakou L., Fernández J.M., Navarro-Blasco Í., Álvarez J.I. (2026). Evaluating the durability and cyclic thermal performance of lime mortars with microencapsulated PCMs for sustainable energy solutions. Constr. Build. Mater..

[46] Lucas S.S., de Aguiar J.L.B. (2019). Evaluation of latent heat storage in mortars containing microencapsulated paraffin waxes—A selection of optimal composition and binders. Heat Mass Transf..

[47] Illampas R., Rigopoulos I., Ioannou I. (2021). Influence of microencapsulated Phase Change Materials (PCMs) on the properties of polymer modified cementitious repair mortar. J. Build. Eng..

[48] (2019). Sustainability of Construction Works. Environmental Product Declarations. Core Rules for the Product Category of Construction Products.

[49] Eurostat (2016). European Road Freight Transport Database.

[50] Persyn D., Lanchas J.D., Jimenez J.B. (2019). Estimating Road Transport Costs Between EU Regions.

[51] Ngo H.T., Kaci A., Kadri E.H., Ngo T.T., Trudel A., Lecrux S. (2017). Energy Consumption Reduction in Concrete Mixing Process by Optimizing Mixing Time. Energy Procedia.

[52] Eurostat (2025). Electricity Prices for Non-Household Consumers—Bi-Annual Data (From 2007 Onwards).

[53] Ismail M., Awang H., Al-Shwaiter A., Al-Absi Z.A., Hafizal M.I.M. (2022). Properties of PCM-based composites developed for the exterior finishes of building walls. Case Stud. Constr. Mater..

[54] González-Sánchez J.F., Fernández J.M., Navarro-Blasco Í., Alvarez J.I. (2021). Improving lime-based rendering mortars with admixtures. Constr. Build. Mater..

[55] Aggelakopoulou E., Bakolas A., Moropoulou A. (2011). Properties of lime-metakolin mortars for the restoration of historic masonries. Appl. Clay Sci..

[56] Su-Cadirci T.B., Calabria-Holley J., Ince C., Ball R.J. (2023). Freeze-thaw resistance of pozzolanic hydrated lime mortars. Constr. Build. Mater..

[57] Duran A., Gonzalez-Sanchez J.F., Fernandez J.M., Sirera R., Navarro-Blasco I., Alvarez J.I. (2018). Influence of Two Polymer-Based Superplasticizers (Poly-naphthalene Sulfonate, PNS, and Lignosulfonate, LS) on Compressive and Flexural Strength, Freeze-Thaw, and Sulphate Attack Resistance of Lime-Metakaolin Grouts. Polymers.

[58] Dinakar P., Sahoo P.K., Sriram G. (2013). Effect of Metakaolin Content on the Properties of High Strength Concrete. Int. J. Concr. Struct. Mater..

[59] Arizzi A., Cultrone G. (2012). Aerial lime-based mortars blended with a pozzolanic additive and different admixtures: A mineralogical, textural and physical-mechanical study. Constr. Build. Mater..

[60] Xu D., Qi G.D., Wang D.M., Zhang D.J., Zhang S. (2025). Competitive mechanisms of hydration and carbonation in hydraulic lime under natural and accelerated carbonation (3% CO_2_). J. Build. Eng..

[61] Zetola V., Claros-Marfil L.J., Santos A.G., González F.J.N. (2021). Effect of Paraffin and Silica Matrix Phase Change Materials on Properties of Portland Cement Mortars. Materials.

[62] Islam M.R., Li X. (2023). Application of from-stable paraffin/nano-silica phase change materials for thermal energy storage in mortar. Int. Adv. Res. J. Sci. Eng. Technol..

[63] Santos A.R., Veiga M.D., Silva A.S., de Brito J., Alvarez J.I. (2018). Evolution of the microstructure of lime based mortars and influence on the mechanical behaviour: The role of the aggregates. Constr. Build. Mater..

[64] Silva A.S., Gameiro A., Grilo J., Veiga R., Velosa A. (2014). Long-term behavior of lime-metakaolin pastes at ambient temperature and humid curing condition. Appl. Clay Sci..

[65] Arizzi A., Viles H., Cultrone G. (2012). Experimental testing of the durability of lime-based mortars used for rendering historic buildings. Constr. Build. Mater..

[66] Grubesa I.N., Markovic B., Vracevic M., Tunkiewicz M., Szenti I., Kukovecz A. (2019). Pore Structure as a Response to the Freeze/Thaw Resistance of Mortars. Materials.

[67] Mahedi M., Cetin B., Cetin K.S. (2019). Freeze-thaw performance of phase change material (PCM) incorporated pavement subgrade soil. Constr. Build. Mater..

[68] Yuan X.S., Wang B.M., Chen P., Luo T. (2021). Study on the Frost Resistance of Concrete Modified with Steel Balls Containing Phase Change Material (PCM). Materials.

[69] Zheng Z.T., Shen W.B., Li S., Li C., Yin P.J., Guan M.J., Ha J. (2025). Mechanical properties and Freeze-Thaw durability of concrete modified with microencapsulated phase change materials. Sci. Rep..

[70] Shi Y., Wang Y., Wang L.-N., Wang W.-N., Yang T.-Y. (2025). Bridge Cable Performance Warning Method Based on Temperature and Displacement Monitoring Data. Buildings.

[71] Shi Y., Wang Y., Wang L.-N., Wang W.-N., Yang T.-Y. (2025). Bridge Tower Warning Method Based on Improved Multi-Rate Fusion Under Strong Wind Action. Buildings.

[72] Yan Y., King S.C., Li M., Galy T., Marszewski M., Kang J.S., Pilon L., Hu Y.J., Tolbert S.H. (2019). Exploring the Effect of Porous Structure on Thermal Conductivity in Templated Mesoporous Silica Films. J. Phys. Chem. C.

[73] An L., Di Luigi M., Petit D., Hu Y., Chen Y.J., Armstrong J.N., Li Y.G.C., Ren S.Q. (2022). Nanoengineering Porous Silica for Thermal Management. ACS Appl. Nano Mater..

[74] Bose P., Amirtham V.A. (2016). A review on thermal conductivity enhancement of paraffinwax as latent heat energy storage material. Renew. Sustain. Energy Rev..

[75] Mitran R.A., Ionita S., Lincu D., Berger D., Matei C. (2021). A Review of Composite Phase Change Materials Based on Porous Silica Nanomaterials for Latent Heat Storage Applications. Molecules.

[76] Himran S., Suwono A., Mansooru G.A. (1994). Characterization of alkanes and paraffin waxes for application as Phase-Change Energy-Storage medium. Energy Sources.

[77] Ordonez-Miranda J., Alvarado-Gil J. (2012). Effect of the pore shape on the thermal conductivity of porous media. J. Mater. Sci..

[78] Yu Z.M., Huang Y.M., Dong W., Zhao X.K., Wang F., Ma G.W. (2026). Pore distributive heterogeneity of cementitious materials by poker vibration: Quantification and modification. Case Stud. Constr. Mater..

[79] Li Y., Dong M.Y., Song W., Liang X.Y., Chen Y.W., Liu Y.F. (2021). Preparation and Characterization of Paraffin/Mesoporous Silica Shape-Stabilized Phase Change Materials for Building Thermal Insulation. Materials.

[80] Peng H., Zhang D., Ling X., Li Y., Wang Y., Yu Q.H., She X.H., Li Y.L., Ding Y.L. (2018). n-Alkanes Phase Change Materials and Their Microencapsulation for Thermal Energy Storage: A Critical Review. Energy Fuels.

[81] Katish M., Allen S., Squires A., Ferrandiz-Mas V. (2024). Thermal stability of organic Phase Change Materials (PCMs) by accelerated thermal cycling technique. Thermochim. Acta.

[82] Sam M.N., Caggiano A., Mankel C., Koenders E. (2020). A Comparative Study on the Thermal Energy Storage Performance of Bio-Based and Paraffin-Based PCMs Using DSC Procedures. Materials.

[83] Rao Z.H., Zhang G.Q. (2011). Thermal Properties of Paraffin Wax-based Composites Containing Graphite. Energy Sources Part A Recovery Util. Environ. Eff..

[84] Mhike W., Focke W.W., Mofokeng J.P., Luyt A.S. (2012). Thermally conductive phase-change materials for energy storage based on low-density polyethylene, soft Fischer–Tropsch wax and graphite. Thermochim. Acta.

[85] Said A., Salah A., Fattah G.A. (2017). Enhanced Thermo-Optical Switching of Paraffin-Wax Composite Spots under Laser Heating. Materials.

[86] Fenrych J., Reynhardt E.C., Basson I. (1997). Structures and molecular dynamics of binary mixtures of n-alkanes: C-46:C-38 and C-46:C-44. Chem. Phys. Lett..

[87] El Rhafiki T., Kousksou T., Jamil A., Jegadheeswaran S., Pohekar S.D., Zeraouli Y. (2011). Crystallization of PCMs inside an emulsion: Supercooling phenomenon. Sol. Energy Mater. Sol. Cells.

[88] Kyriakou L., Rubio-Aguinaga A., Nofalah M.H., Paz L.M.P., Molino Á.G., Ferrara L., Karatasios I., Tziviloglou E., Fernández J.M., Navarro-Blasco Í. (2026). From laboratory formulation to in situ evaluation: PCM-enhanced lime-pozzolan-cement mortars for thermal retrofit of heritage architecture. Dev. Built Environ..

[89] Ansari J.A., Al-Shannaq R., Kurdi J., Al-Muhtaseb S.A., Ikutegbe C.A., Farid M.M. (2021). A Rapid Method for Low Temperature Microencapsulation of Phase Change Materials (PCMs) Using a Coiled Tube Ultraviolet Reactor. Energies.

[90] Sarcinella A., Cunha S., Aguiar I., Aguiar J., Frigione M. (2025). Sustainable Organic Phase Change Materials for Sustainable Energy Efficiency Solutions. Polymers.

[91] Huang Y.C., Stonehouse A., Abeykoon C. (2023). Encapsulation methods for phase change materials-A critical review. Int. J. Heat Mass Transf..

[92] Habert G., de Lacaillerie J.B.D., Roussel N. (2011). An environmental evaluation of geopolymer based concrete production: Reviewing current research trends. J. Clean. Prod..

[93] Pacheco-Torgal F., Jalali S. (2011). Nanotechnology: Advantages and drawbacks in the field of construction and building materials. Constr. Build. Mater..

[94] Guardia C., Barluenga G., Palomar I. (2020). PCM Cement-Lime Mortars for Enhanced Energy Efficiency of Multilayered Building Enclosures under Different Climatic Conditions. Materials.

[95] United Nations Environment Programme (2022). Global Status Report for Buildings and Construction 2022: Towards a Zero-Emission, Efficient and Resilient Buildings and Construction Sector.

[96] (2025). Nearly-Zero Energy and Zero-Emission Buildings. Energy Performance of Buildings Directive (EPBD) Recast—Directive.

[97] Fei W.M., Opoku A., Agyekum K., Oppon J.A., Ahmed V., Chen C., Lok K.L. (2021). The Critical Role of the Construction Industry in Achieving the Sustainable Development Goals (SDGs): Delivering Projects for the Common Good. Sustainability.

