# Supplementary material for: Multi-PCM Lime Mortars Incorporating Polymer-Shell and Form-Stable Phase Change Materials for Energy-Efficient Building Envelopes
Source: Polymers (Basel). 2026 Jun 12;18(12):1481. doi: 10.3390/polym18121481 (PMC13307531; doi:10.3390/polym18121481)
Supplement: Supplementary file 1 [file polymers-18-01481-s001.zip › polymers-4359579-supplementary.pdf]

SUPPLEMENTARY MATERIAL

Multi-PCM lime mortars incorporating polymer-shell and form-stable Phase Change Materials for energy-efficient building envelopes renders

A. Rubio-Aguinaga[a], L. Kyriakou[a], J.M. Fernández[a], I. Navarro-Blasco[a], J.I. Álvarez[a*]

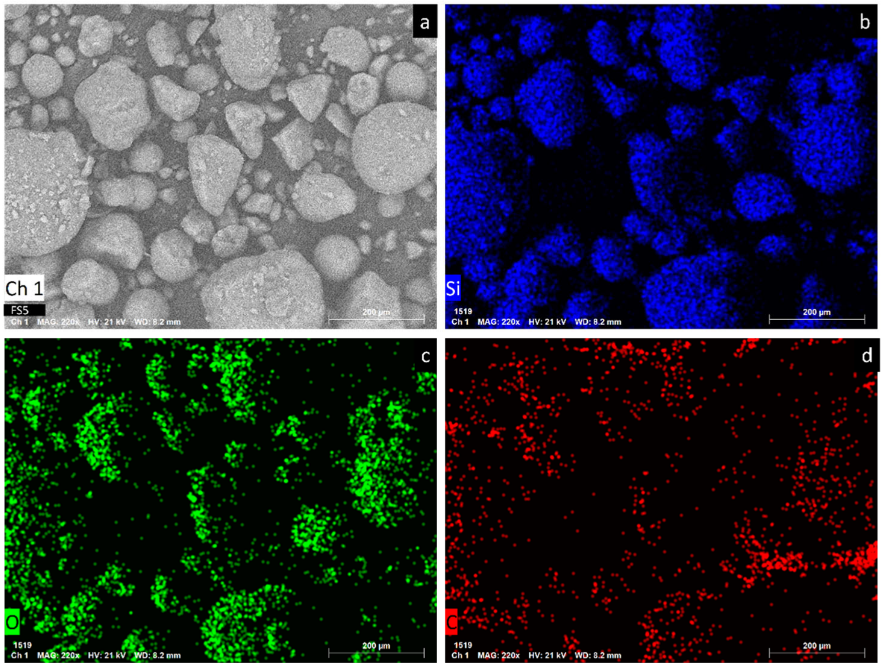

Figure S1. SEM micrograph and EDS elemental maps of the pure form-stable PCM FS5: (a) SEM micrograph, (b) Si elemental map, (c) O elemental map and (d) C elemental map.

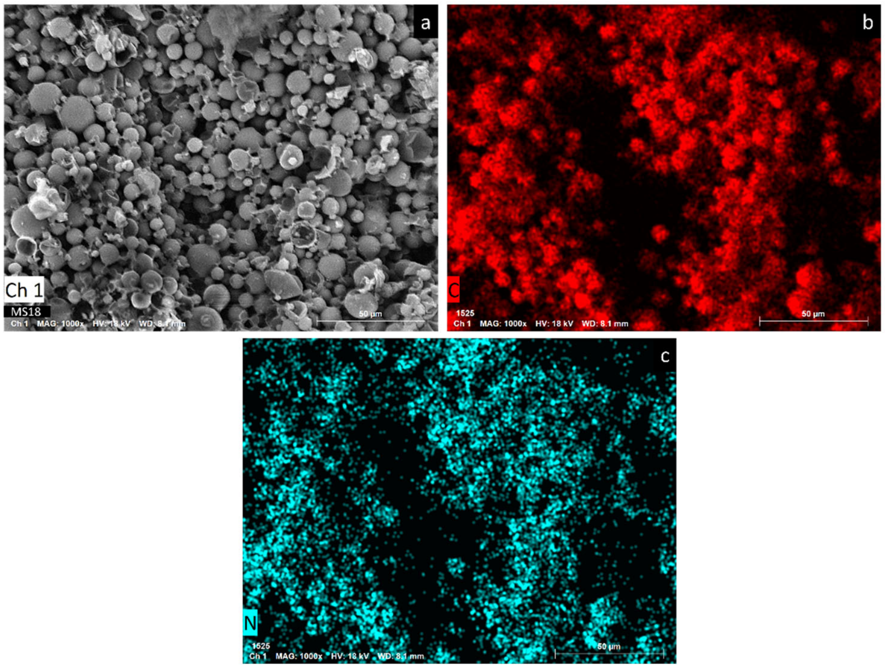

Figure S2. SEM micrograph and EDS elemental maps of the pure slurry-microencapsulated PCM MS18: (a) SEM micrograph, (b) C elemental map and (c) N elemental map.

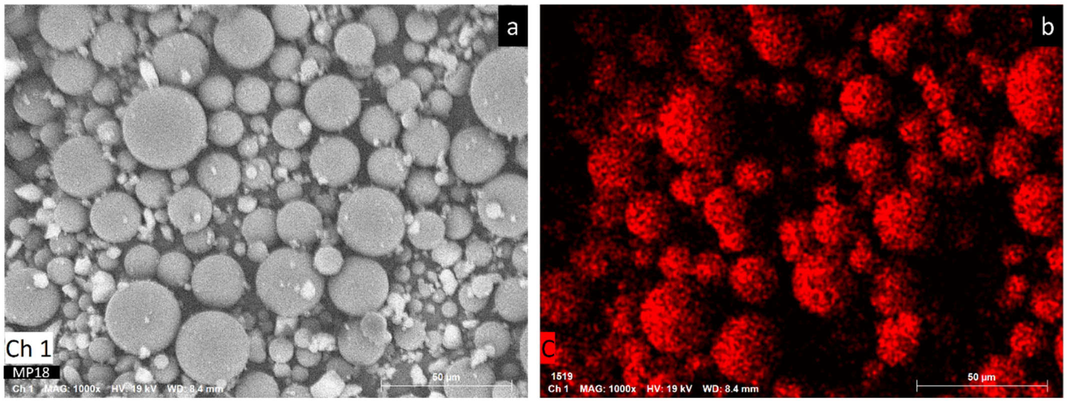

Figure S3. SEM micrograph and EDS elemental map of the pure powder-microencapsulated PCM MP18: (a) SEM micrograph and (b) C elemental map.

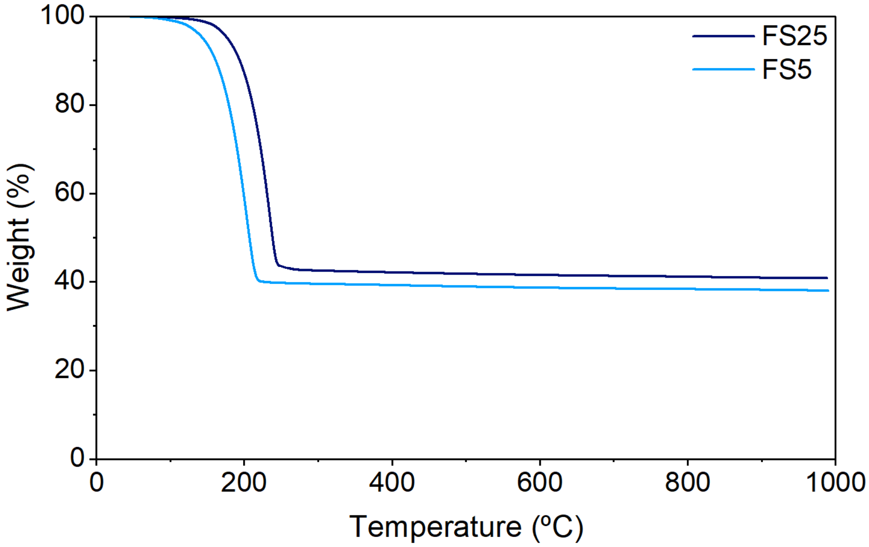

Figure S4. TGA curves of the pure form-stable PCMs: FS25 and FS5.

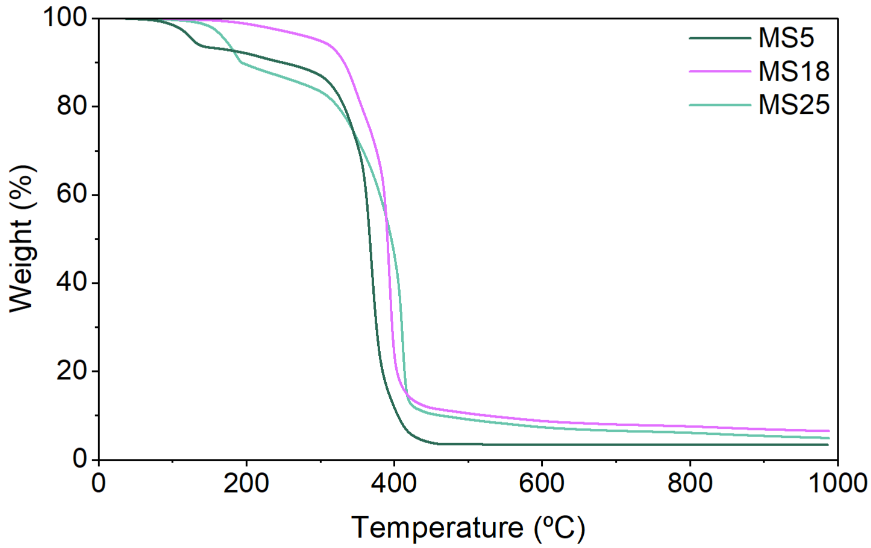

Figure S5. TGA curves of the pure slurry-microencapsulated PCMs: MS5, MS18 and MS25.

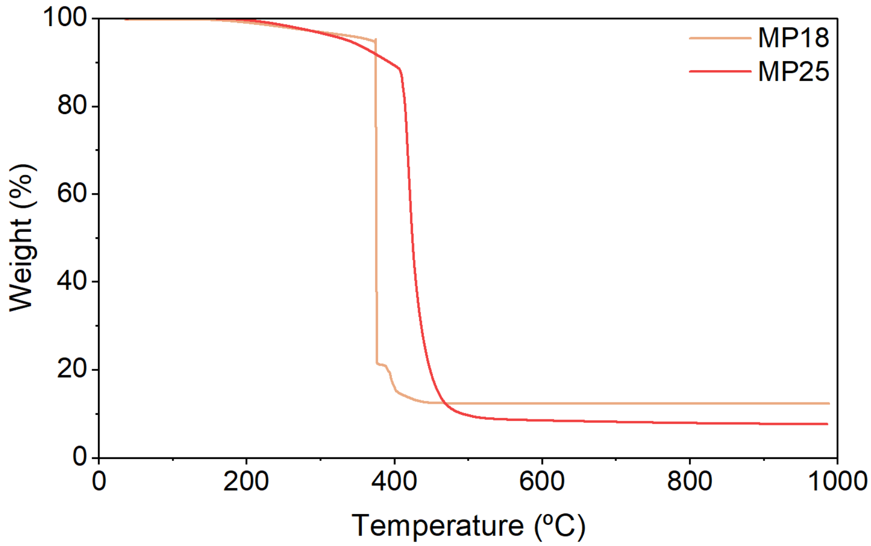

Figure S6. TGA curves of the pure powder-microencapsulated PCMs: MP18 and MP25.
